# Comparative transcriptome analysis provides global insight into gene expression differences between two orchid cultivars

**DOI:** 10.1371/journal.pone.0200155

**Published:** 2018-07-05

**Authors:** Yu Jiang, Hai-Yan Song, Jun-Rong He, Qiang Wang, Jia Liu

**Affiliations:** 1 College of Agronomy, Sichuan Agricultural University, Chengdu, Sichuan Province, China; 2 Institute of Horticulture, Sichuan Academy of Agricultural Science, Chengdu, Sichuan Province, China; Youngstown State University, UNITED STATES

## Abstract

The orchids GL and YL are two cultivars of *Cymbidium longibracteatum*. YL displays an obviously yellowing rhizome and yellow leaves, while GL ('Longchangsu') shows dark green leaves and greenish rhizome. But the molecular mechanism for the differences between the two cultivars is poorly understood. In the present study, we showed that the structure of chloroplasts was significantly damaged in YL. Biochemical analysis uncovered the contents of chlorophyll a, chlorophyll b, total chlorophyll and carotenoid were notably decreased in YL. Using RNA-Seq technology, more than 38 million clean reads were generated in each pool, and 116,422 unigenes were assembled *de novo*. 6,660 unigenes with differential expression patterns (FDR≤0.01 and |log2 ratio|≥1) were totally identified between the two cultivars. Kyoto Encyclopedia of Genes and Genomes (KEGG) analysis of differentially expressed unigenes (DEGs) suggested 33 KEGG pathways were notably enriched, including biological processes such as “phenylpropanoid biosynthesis”, “phagosome”, “starch and sucrose metabolism”, “drug metabolism—cytochrome P450”, “fatty acid elongation”, and “flavone and flavonol biosynthesis”. Further analysis revealed that chlorophyll degeneration related unigene (c48794_g1) and flavonoid biosynthesis related unigenes (c16388_g1, c48963_g1, c63571_g1, c4492_g1, c52282_g1, c78740_g1, c4645_g1) were up-regulated while carotenoid biosynthesis related unigene (c7212_g1) were down-regulated in YL. Additionally, six of NAC, R2R3-MYB, bHLH transcription factors (c42861_g1, c105949_g1, c61265_g1, c42659_g1, c82171_g1, c19158_g1) might be involved in regulation of pigment biosynthesis. The chlorophyll degeneration and the flavonoid biosynthesis related unigenes up-regulation together with the carotenoid biosynthesis related unigenes down-regulation may contribute to the yellowing phenotype of YL.

## Introduction

Orchidaceae is one of two largest families of flowering plants. It has approximately 26,000 species distributed in approximately 880 genera around the world [1]. *Cymbidium* is an important economical genus of Orchidaceae because of its beautiful flower posture, and it acts as a cultural symbol in many regions around the world, especially East Asia [2]. In general, Chinese orchids are widely cultivated in China and include seven species of the genus *Cymbidium*: *C*. *goeringii*, *C*. *faberi*, *C*. *ensifolium*, *C*. *sinense*, *C*. *kanran*, *C*. *1ianpan* and *C*. *longibracteatum* [3]. Because people constantly pursue novelty, wild orchid bud-mutation cultivars do not meet global flower market requirements. Recently, biotechnological methods, such as tissue culture-induced genetic mutation, have been widely used to create new cultivars [4]. Using this method, we generated a new orchid cultivar (abbreviated as YL in this study) that can be easily distinguished from wild-type *C*. *longibracteatum* 'Longchangsu' (abbreviated as GL in this study) [5]. In contrast with GL having dark green leaves and greenish rhizome, YL is some dwarf with an obviously yellowing rhizome and yellow leaves. However, the molecular mechanisms for the differences between the two cultivars are poorly understood.

The genomes of Orchidaceae vary greatly among different genera. Only several genera genomes have been recently sequenced in past [6, 7]. RNA-Seq is a revolutionary tool for exploring genetic information, especially in non-model organisms [8]. Recently, some works on orchids have been explored by RNA-Seq [9–11], and several key genes or pathways underpinning the processes are further revealed [12–14]. (S)-b-citronellol and 2-hydroxy-6-methylacetophenone are two active semiochemicals in *Caladenia plicata* that mimic female sex pheromones lure their specific male pollinators. By comparative transcriptome analysis of contrasting floral tissue, Xu et al identify the key genes that involve the biosynthesis of (S)-b-citronellol and 2-hydroxy-6-methylacetophenone [12]. Chiloglottone 1 is a unique UV-B-dependent floral volatile of *Chiloglottis trapeziformis* for specific male wasp pollinator attraction. Wong et al reveal the pathway of chiloglottone 1 biosynthesis by deep transcriptome sequencing [13]. In the present study, the biochemical, cytological and comparatively transcriptomical analysis of YL and GL were systematically performed, and several key genes related to the pigments change between two cultivars were detected. These findings will shed light on our understanding of the differences in the molecular mechanisms between two cultivars and will help in future breeding of the orchids.

## Materials and methods

### Plant materials

YL and GL were generated by tissue culture method [5], and grown for one year under controlled temperatures of 25/20°C (day/night) in mericlone nursery at the Horticulture Institute of Sichuan Agricultural Sciences in Chengdu city, Sichuan province, P. R. China. For each cultivar, three random plants were selected for analysis. The youngest five leaves from each plant were collected and flash-frozen in liquid nitrogen for RNA isolation, and the remaining plantlets were used for biochemical analysis and electron microscopy.

### Transmission electron microscopy

Leaf samples from the remaining plantlet for each cultivar were sliced into 2.0-mm^2^ sections and pre-fixed in 2% glutaraldehyde. The samples were incubated at 4°C in a refrigerator overnight and then rinsed three times with double distilled water. The prefixed tissues were stained with 1% osmium tetroxide for 30 minutes and then rinsed four times in double distilled water to remove any unbound osmium tetroxide. The stained and washed tissues were dehydrated with ethanol and acetone and embedded in an epoxy resin Epon812 following the manufacturer’s instructions. Ultra-thin sections of the embedded leaf were sliced using a microtome, and ultrastructure was visualized using a JEM-1200 EX transmission electron microscope (Hitachi) according to the instructions described by Hu et al [15].

### Chlorophyll and carotenoid determination

The chlorophyll and carotenoid contents were determined according to the method [16]. Fresh leaves (0.1 g) from YL and GL were homogenized in 10 mL of 80% acetone. Then, the homogenate was centrifuged at 12,000 rpm for 5 min, and the supernatant was used for measurement at 663 nm (chlorophyll a), 645 nm (chlorophyll b) and 440 nm (carotenoid). The chlorophyll and carotenoid contents were measured with three replicates.

### RNA isolation and cDNA library construction

Total RNA was isolated using the TRIzoI reagent (Plant RNA Purification Reagent, Invitrogen) according to the manufacturer’s protocol. A Nanodrop 2000 was used to detect the concentration and purity of the total RNA, while the integrity was verified by agarose gel electrophoresis. The RIN (the minimum integrity number) value of RNA was determined using an Agilent2100 Bioanalyzer (Agilent Tech., CA). The total RNA of three plant per cultivar was pooled in equal amounts for transcriptome sequencing. Oligo-dT magnetic beads (DynaMag-2 Magnet 12321D, Invitrogen) were used to purify poly (A+) mRNA from the total RNA pool. Equal mRNA from three plants was pooled to prepare a cDNA library for each cultivar. The resulting cDNA libraries were sequenced using the Illumina HiSeq 2000 at the Shanghai Major Biological Medicine Technology Co., Ltd. (Shanghai, China).

### *De novo* assembly and annotation

Raw reads were processed by trimming adapter sequences and removing reads with 10% or more uncalled bases and that were shorter than 20 bp as well as low-quality reads from the raw data. The *de novo* assembly of the cleaned reads was performed using Trinity [17] with min_kmer_cov set to 2 and all other parameters set to default. After assembly, the longest transcript from each locus was selected as a unigene for subsequent annotation. The assembled unigenes were annotated by comparison with the proteins in the NCBI’s non-redundant (NR) protein, String, Kyoto Encyclopedia of Genes and Genomes (KEGG), Swiss-Prot and Pfam databases, Clusters of Orthologous Group (COG) database, using BLAST with an E-value < 10–5. The annotation assignment from the protein databases was prioritized as NR, NT, Swiss-Prot, String, Pfam, KEGG and COG.

### Identification of DEGs

Differential expression analysis of the two samples was performed using edgeR [18]. Briefly, p-value of unigenes was calculated by comparative analysis of transcriptome data of GL versus YL. P values were adjusted to obtain the false discovery rate (FDR) using the Benjamini-Hochberg approach [19]. Unigenes with FDR≤0.05 and |log2 ratio|≥1 were considered differentially expressed. KEGG enrichment analysis of the DEGs was performed by the KOBAS software [20].

### Quantitative real-time PCR (qRT-PCR) analysis

Three individual plants of each orchid cultivar were used for qPCR validation. The total RNA from the leaves of each plant was isolated using a Qiagen RNA plant mini kit with on-column DNAse digestion (Qiagen, Inc., CA). Six hundred nanograms of total RNA was reverse transcribed using the Primescript RT reagent kit with gDNA Eraser (Takara Bio Inc., Japan). The cDNA diluted to 200 ng/μL was used for the qRT-PCR assay for each gene with a gene-specific primer pair and SsoFast Eva-Green Supermix (Bio-Rad Laboratories, Inc., CA) on a Bio-Rad CFX96 real-time system. Reactions were performed at 95°C for 3 min followed by 40 cycles at 95°C for 10 s and 58°C for 30 s. The relative abundance of transcripts was calculated according to the 2^-∆∆Ct^ method and normalization with *ACTIN* [21]. All primer pairs used in this article are listed in S1 Table.

### Phylogenetic tree construction

The coding sequence of *AtNAC*, *AtR2R3-MYB*, *AtbHLH* were acquired from the TAIR (http://www.arabidopsis.org/) databases. Other pigment biosynthesis related sequences were obtained from NCBI based on the GenBank accession number (S2 Table). The sequences were aligned using online software Multiple Sequence Alignment (https://www.ebi.ac.uk/Tools/msa/), and then constructed the phylogenetic tree using iTOL online software (http://itol.embl.de/).

### Statistical analysis

Statistical analysis was performed with GraphPad Prism 5 based on the T-test. * and ** represent 0.05 and 0.01 significant differences, respectively. Correlation analysis was performed with SPSS 22.0. The heatmap of unigenes was performed using Genesis software.

## Results

### Difference in cytology and physiology between YL and GL

**Fig 1:**
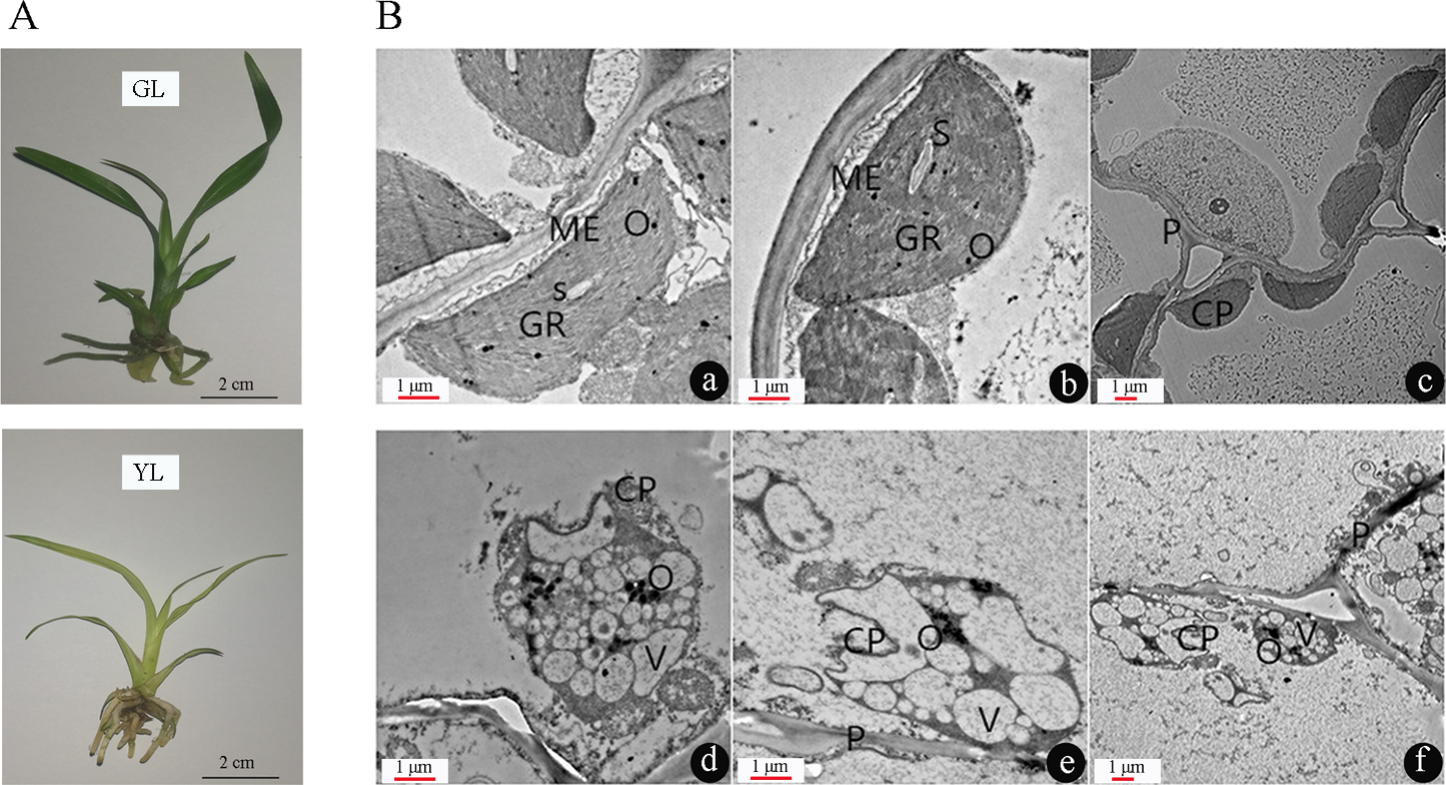
**Phenotypes (A) and chloroplast ultrastructures (B) of GL and YL.** a-c: chloroplast ultrastructures of GL. d-f: chloroplast ultrastructures of YL. CP: chloroplast; GR: granum; S: starch grain; O: osmiophile globule; V: vesicle; ME: membrane envelops of chloroplast; P: plasmodesmata.

There are obvious phenotypic differences between YL and GL. GL shows dark green leaves and greenish rhizome, while YL is some dwarf with an obviously yellowing rhizome and yellow leaves (Fig 1A). Cytological observation of the chloroplasts revealed a large structural change between the two cultivars. The chloroplasts of GL had an intact thylakoid structure, grana layers arranged along the long axis, starch grains and osmiophilic droplets dispersed in the matrix. In contrast, the chloroplast structure in YL was severely damaged. The osmiophilic droplets were aggregated, while the starch grains were disappeared (Fig 1B).

Chlorophyll and carotenoid determination showed that there were significantly changed between the two cultivars (Table 1). The chlorophyll a, b and total chlorophyll contents in YL were approximately 83% lower than in GL. Similarly, the content of carotenoid was decreased by 79% in YL. However, the ratios of chlorophyll a / chlorophyll b and carotenoid / total chlorophyll showed increase in YL.

**Table 1:** Content of chlorophyll and carotenoids in YL and GL.

Plants	Chl a (mg.g^-1^)	Chlb (mg·g^-1^)	Chl a+b (mg.g^-1^)	Chl a / Chl b	Carotenoids (mg.g^-1^)	Carotenoid / Chl
**GL**	8.58±.58	3.06±.05	11.64	2.81	3.048±.05	0.26
**YL**	1.43±.43^**^	0.49±.49^**^	1.92^**^	2.95^*^	0.631±.63^**^	0.33^*^

* and ** represent 0.05 and 0.01 significant differences, respectively.

### Sequencing, assembly and annotation of *C*. *longibracteatum* transcriptome

**Fig 2:**
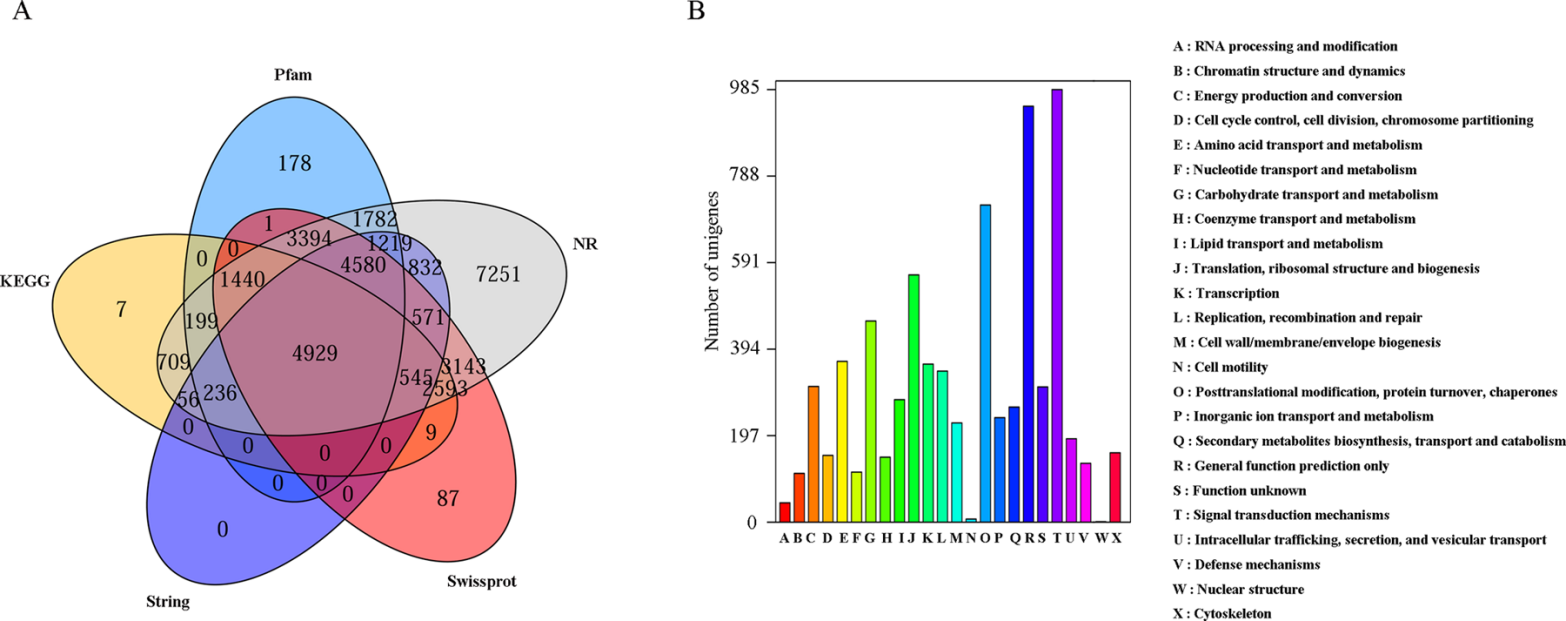
Transcriptome annotation and COG classifications of assembled unigenes. (A) Transcriptome annotation against NR database, String, KEGG, UniProt/Swiss-Prot, Pfam databases. (B) COG classifications of assembled unigenes.

RNA from the leaves of YL and GL were extracted and sequenced using the Illumina HiSeq 2000 platform. After filtering and quality trimming the raw reads, 39,557,830 and 38,536,724 high-quality reads (clean reads) were obtained for each cultivar, respectively (NCBI accession number: GSE100180) (S3 Table). Finally, a total of 116,422 unigenes with an average length of 598 bp and an N50 of 896 bp were assembled *de novo* (see S1 File). Under the E-value threshold of 1E-5, the unigenes were used to match against the publicly available database, such as the NR database, String, KEGG, UniProt/Swiss-Prot, and Pfam databases. It was shown that 33,487 (28.77%), 12,968 (11.14%), 10,723 (9.21%), 21,297 (18.30%) and 17,958 (15.43%) unigenes were matched, respectively (Fig 2A). COG annotation of transcriptome can reveal the main function of a unigene and its probable evolutionary affinities [22]. In the present research, 7,426 unigenes could be divided into 24 COG categories (Fig 2B). The cluster for “signal transduction mechanisms” represented the largest group (985 unigenes), followed by “general function prediction only” (947 unigenes), and “posttranslational modification, protein turnover, chaperones” (722 unigenes). Nevertheless, categories of “nuclear structure” and “cell motility” only included 1 and 7 unigenes.

### Analysis of DEGs

The unigenes expression was obtained by comparative analysis of transcriptome data of GL versus YL. FDR was obtained by adjusting P-value using the Benjamini-Hochberg method. The criteria of FDR ≤ 0.01 and |log2 ratio| ≥ 1 is generally used in DEGs detection in RNA-Seq [23– 24]. Under the criteria, 6,660 unigenes with differential expression (4,543 up-regulation and 2,117 down-regulation) were identified by comparative analysis of transcriptome data of GL versus YL (see S2A File). “Up-regulation” indicates that the transcript of the unigene was abundant in YL, while “down-regulation” indicates that the transcript of the unigene was abundant in GL. To validate the expression of DEGs, 10 unigenes were selected for amplification by qRT-PCR in the leaves of YL and GL. The results showed that the expression pattern of most of them was similar to the digital gene expression data (see S2B File).

### KEGG functional enrichment analysis of the DGEs

To better understand the molecular difference between YL and GL, the up-regulated and down-regulated DGEs were grouped by KEGG. 6,660 DGEs were grouped into 76 KEGG pathways, and 33 of them showed significant enrichment (p value < 0.05), including “phenylpropanoid biosynthesis” (p value: 1.41E-08), “starch and sucrose metabolism” (p value: 3.23E-07), “drug metabolism—cytochrome P450” (p value: 0.0065), “fatty acid elongation” (p value: 0.0054), “flavone and flavonol biosynthesis” (p value: 0.0026) and “phagosome” (p value: 0.002) (see S3 File).

### Differences in expression patterns of pigment-related unigenes between YL and GL

**Fig 3:**
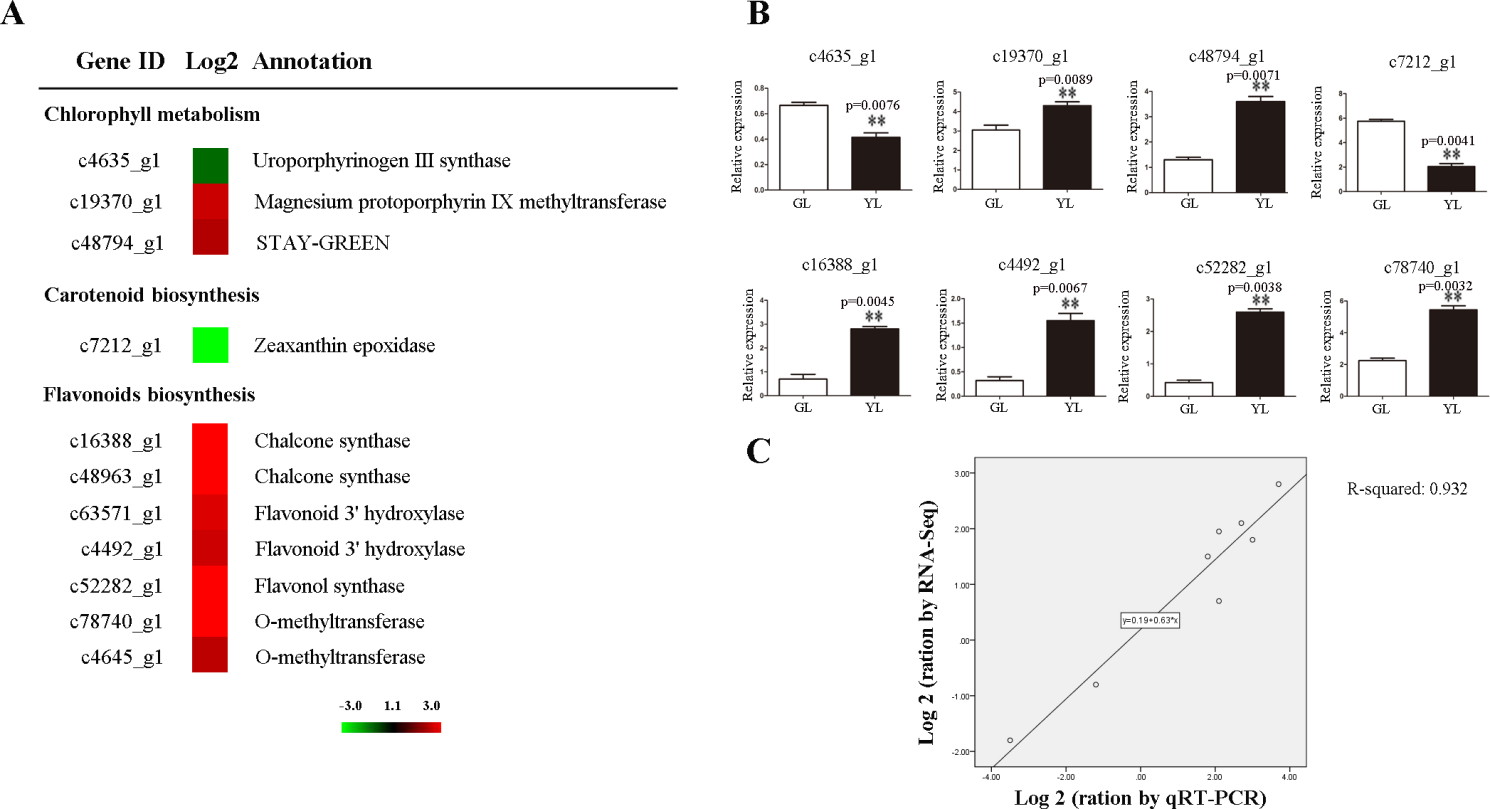
Differential expressed unigenes involving in pigment metabolism. The left showed heat map of unigenes (A) and the right showed qRT-PCR verification (B-C). DGE values displayed as heat map. Colours bar represented expression levels of each unigene which were either up-regulated (red) or down-regulated (green). Error bars for qRT-PCR showed the standard deviation of three replicates. ** represented 0.01 significant difference. Correlation analysis of Log2 change values obtained from RNA-Seq and qRT-PCR.

Chlorophyll, carotenoids and flavonoids are the main plant pigments and contribute to leaf colour [25]. In the present study, three unigenes involved in chlorophyll metabolism were detected (Fig 3A). c19370_g1, encoding magnesium protoporphyrin IX methyltransferase, and c48794_g1, encoding STAY-GREEN protein, were up-regulated in YL, while c4635_g1, encoding uroporphyrinogen III synthase, was up-regulated in GL. Moreover, c7212_g1, encoding zeaxanthin epoxidase, a key enzyme in carotenoid biosynthesis, was notably down-regulated in YL (Fig 3A). Flavonoids are a class of secondary metabolites, and many are coloured compounds. In the current study, unigenes encoding chalcone synthase (c16388_g1 and c48963_g1), flavonoid 3' hydroxylase (c63571_g1 and c4492_g1), flavonol synthase (c52282_g1), and O-methyltransferase (c4645_g1 and c78740_g1) were identified, and all of them were up-regulated in YL (Fig 3A). The expression levels of eight unigenes were further confirmed by qRT-PCR (Fig 3B and 3C).

**Fig 4:**
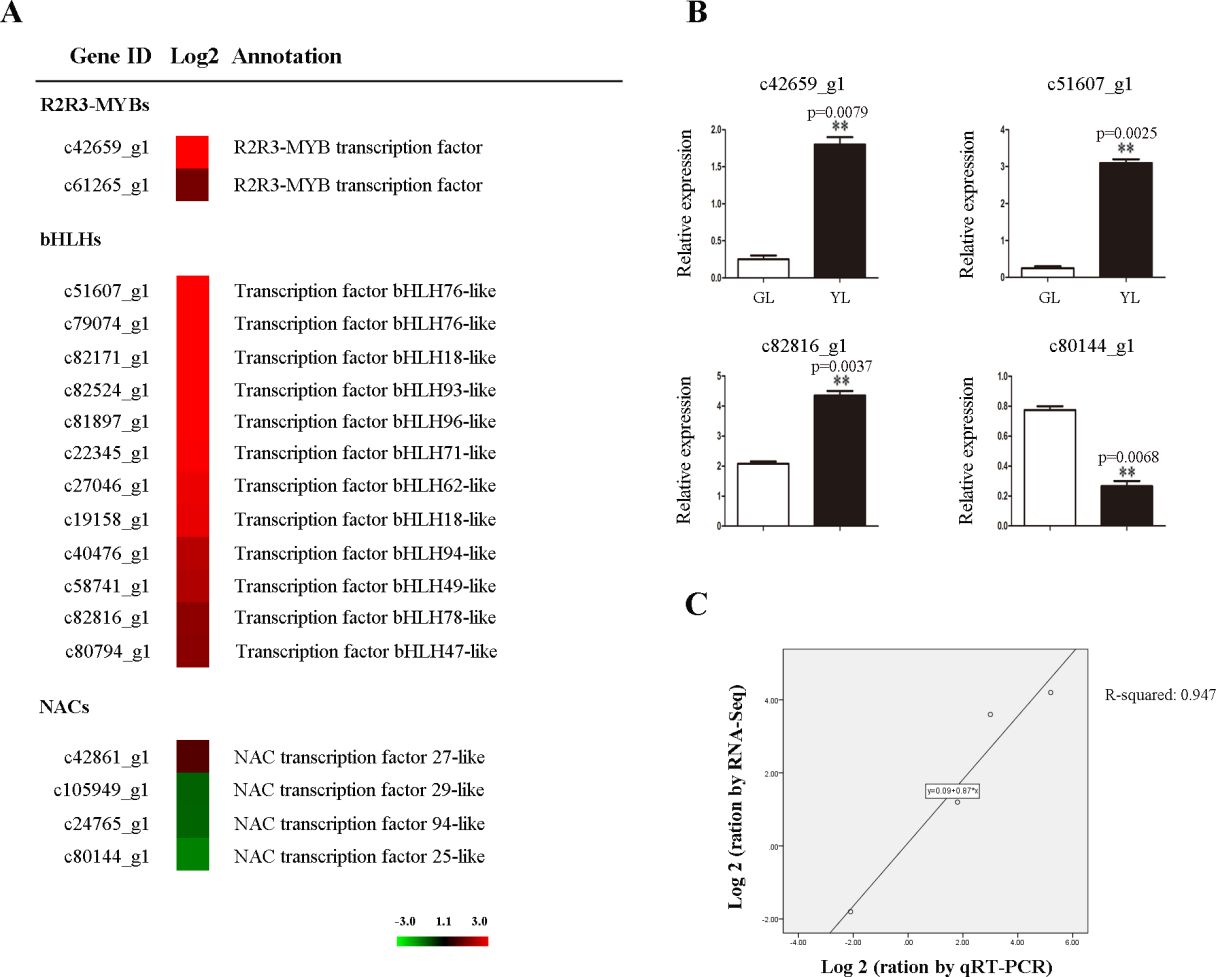
Differential expressed unigenes of transcription factors related to pigment metabolism. The left showed heat map of unigenes (A) and the right showed qRT-PCR verification (B-C). DGE values displayed as heat map. Colours bar represented expression levels of each unigene which were either up-regulated (red) or down-regulated (green). Error bars for qRT-PCR showed the standard deviation of three replicates. ** represented 0.01 significant difference. Correlation analysis of Log2 change values obtained from RNA-Seq and qRT-PCR.

Transcription factors such as R2R3-MYBs, bHLHs, NACs have been shown to regulate the biosynthesis of pigment in plant [26, 27]. Here, two unigenes (c42659_g1, c61265_g1) encoding R2R3-MYBs and twelve unigenes (c19158_g1, c27046_g1, c40476_g1, c51607_g1, c82524_g1, c22345_g1, c58741_g1, c79074_g1, c82816_g1, c82171_g1, c81897_g1, c80794_g1) encoding bHLHs were up-regulated in YL (Fig 4A). In addition, four unigenes (c105949_g1, c42861_g1, c80144_g1, c24765_g1) encoding NACs were detected, and three of them were down-regulated in YL (Fig 4A). The expression levels of four unigenes were further confirmed by qRT-PCR (Fig 4B and 4C).

## Discussion

The orchids GL and YL are two cultivars of *C*. *longibracteatum*, but the molecular mechanism for the phenotypic differences between the two cultivars is poorly understood. In the present research, a systematic analysis of biochemistry, cytology and comparative transcriptome between two cultivars are performed. Though the samples of each cultivar for RNA-Seq are respectively pooled, the genes of key pathways are further validated using independent biological samples of each cultivar, and the expression pattern of genes is consistent with both DEGs and the change of secondary metabolites content (see S2–S3 File and Table 1), suggesting the data is meaningful in some way. To ensure the reliability of DE results, more biological replicates will be performed in further transcriptome research on *Cymbidium*. Orchistra2.0 is a powerful tool that integrates mass of previously reported Orchist transcriptome [28]. To deeply understand the information of DEGs, some unigenes related to pigments metabolism are further explored by comparing with *C*. *sinense* and *C*. *ensifolium* databases of Orchistra2.0 (S4 Table), and the sequences are listed as a additional file (S5 Table). The transcriptome provides an important resource not only for the investigation of molecular mechanism involved in phenotypic differences between two cultivars but also for the molecular research on *Cymbidium*, considering the lack of the genome sequence currently (S4 and S1 Tables).

Cytological observation demonstrates the structure of chloroplasts was significantly damaged in YL (Fig 1). Functional analysis of the DGEs shows mass of biological processes are reprogrammed between the two cultivars (see S3 File). Phagosomes are vesicles formed around foreign particles, such as solid materials, microbes, and cell debris, which play a key role in cell defence and tissue development [29]. Using an autophagy induction system, Lin et al [30] showed that organelles are severely degraded in transgenic *Arabidopsis*, accompanied by the emergence of more autophagosomes. In the present research, we show that the structure of chloroplasts is collapsed in YL (Fig 1). To avoid harm to the cell, the debris should be removed by specific organelles such as phagosomes. Carbohydrate is the basic material for plant growth and development and is synthesized in chloroplasts [31]. As YL chloroplast structure collapses, it is not surprising that starch and sucrose metabolism are influenced (see S3 File). The phenylpropanoid pathway serves as a rich source of metabolites such as flavonoids, a large family of specific coloured metabolites in plant [32, 33]. CHS catalyzes the first step of flavonoid biosynthesis by directing carbon flux from phenylpropanoid metabolism [34]. In tomato, suppression of CHS expression can significantly reduce the content of flavonoids in cuticle [35]. The notable enrichment of phenylpropanoid metabolism may contribute to the rhizome and leaves colour difference between two cultivars, as two CHS encoding unigenes (c16388_g1 and c48963_g1) with differential expression (Fig 3). Cytochrome P450 is a subfamily of haemoproteins and can catalyse many biochemical reactions [36]. In plants, P450s are involved in metabolism processes related to pigment formation, defence response, photosynthesis, respiration, etc [37]. In tomato, Zhang et al [38] found that two cytochrome P450s regulate yellow stigma formation by indirectly enhancing the biosynthesis of yellow-coloured naringenin chalcone in the stigma of tomato. Fatty acid metabolism not only produces energy but also provides a carbon skeleton for organic compound formation. Malonyl-CoA (a product of fatty acid metabolism), together with phenylalanine (a product of phenylpropanoid biosynthesis), are the direct precursors for the formation of flavonoids, a class of coloured metabolites in plants [39].

Chlorophyll, carotenoids and flavonoids are the major pigments for plant colour formation. Changes in pigment components alter plant coloration [40]. In the present study, although the contents of chlorophyll and carotenoid are decreased, the ratios of chlorophyll a / chlorophyll b and carotenoid / total chlorophyll are increased in YL (Table 1), suggesting that pigment components are changed between the two cultivars. It is well known that the chloroplast thylakoid membrane is where chlorophyll and carotenoid biosynthesis occur, while flavonoids are synthesized in the cytoplasm [41]. The collapse of chloroplast structure causes the repression of chlorophyll and carotenoid biosynthesis, which why the contents of chlorophyll a, chlorophyll b, total chlorophyll and carotenoid are decreased in YL (Table 1). In the current study, we detected and validated three chlorophyll metabolism-related unigenes and one carotenoid biosynthesis-related unigene with differential expression between the two cultivars (Fig 3). STAY-GREEN protein (SGR, coded by c48794_g1) is a key component of controlling chlorophyll degeneration by destabilizing protein-pigment complexes in the thylakoid membranes [42]. In *Chrysanthemums*, Ohmiya et al [43] show that the expression level of *SGR* is drastically increased in white mutations. Zeaxanthin epoxidase (ZEP) catalyses the downstream reaction of carotenoid biosynthesis [44]. In *Arabidopsis*, mutation of *ZEP* disrupts the epoxidation of zeaxanthin and reduces antheraxanthin, violaxanthin, and neoxanthin levels [45]. We thus propose that the activation of *SGR* and repression of *ZEP* cause a decrease in chlorophyll and carotenoid content in YL. Flavonoids are a group of coloured metabolites such as anthocyanins, aurones, chalcones, flavonols, and proanthocyanidins [46]. In the present study, seven unigenes related to flavonoid biosynthesis are up-regulated (Fig 3), suggesting that flavonoid contents are largely accumulated in YL. This result is in accordance with anthocyanin accumulation in *Anthurium andraeanum* leaf colour mutations [40].

**Fig 5:**
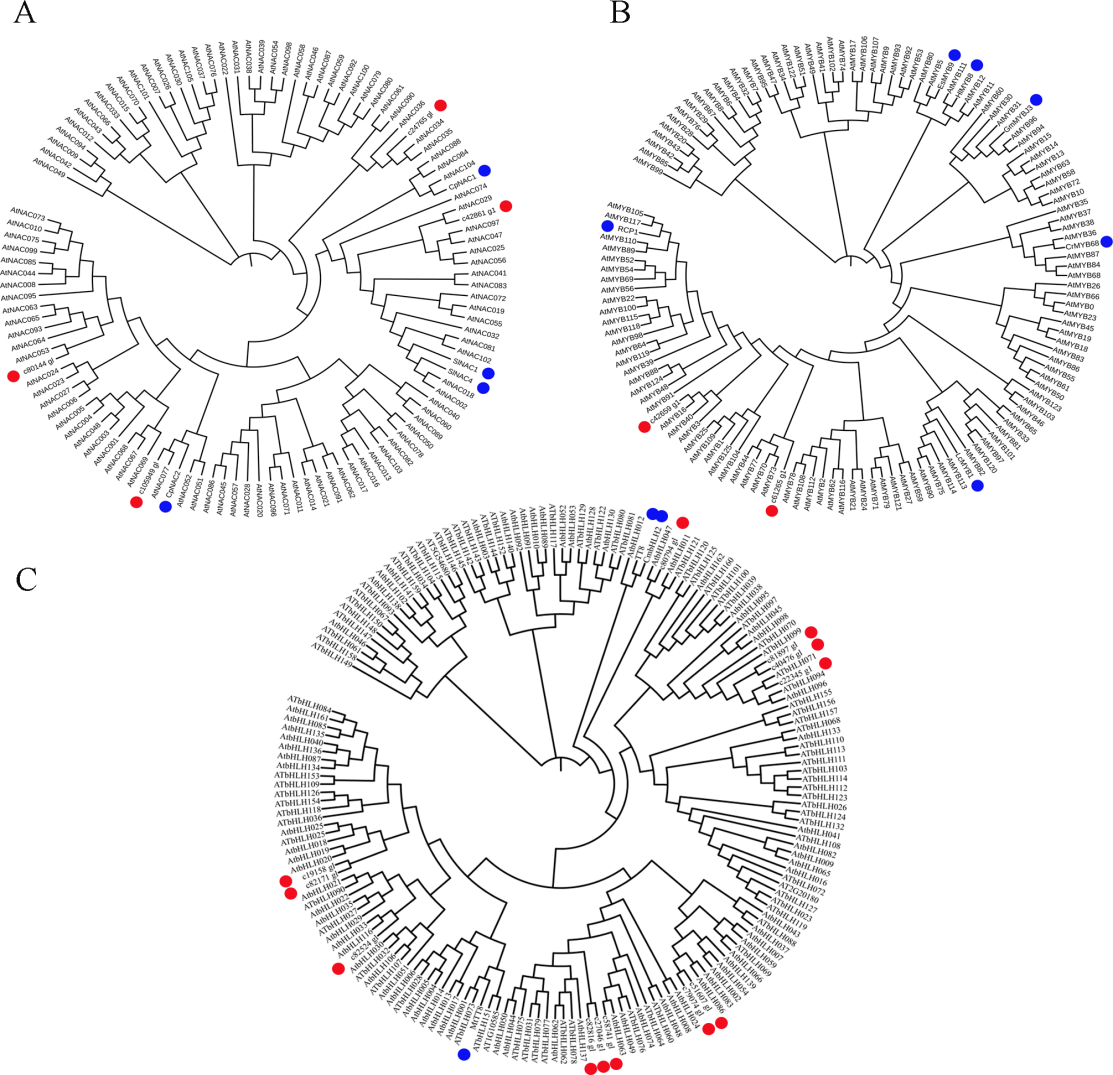
**Phylogenetic relationships of NAC (A), R2R3-MYB (B), bHLH (C) related unigenes with Arabidopsis and other species homologue.** The trees were constructed by neighboring-joining phylogeny test, and 1,000 bootstrap replicates. The accession numbers for the genes are provided in S2 Table. Blue circle represented pigments biosynthesis related TFs in other species. Red circle represented unigenes identified in this study.

Transcription factors such as NACs, R2R3-MYBs, bHLHs, have been identified as the key regulators for pigment biosynthesis [26, 27]. In the present study, 16 unigenes encoding TFs (NAC, R2R3-MYB, bHLH) are detected (Fig 4). To identify putative pigment biosynthesis related members, three phylogenetic trees are further individually constructed by integration of Arabidopsis and other species homologue (Fig 5). NAC TFs represent one of the largest plant-specific transcription factor families, and involve in various plant biological processes such as development, stress responses, senescence, etc [47–49]. In tomato, both suppression of SlNAC4 expression and over-expression of SlNAC1 can reduce the content of carotenoid in transgenic plant [50, 51]. Phytoene desaturase (PDS) is the key enzyme for the conversion of phytoene to ζ-carotene within carotenoid biosynthesis pathway [52]. Fu et al show that both CpNAC1 and CpNAC2 can bind the promoter regain of *CpPDS2/4* [27, 53]. In the present here, phylogenetic analysis shows that c105949_g1 is high similar to CpNAC2, while c42861_g1 together with CpNAC1 and SlNAC1/4 are grouped into same subgroup (Fig 5A), suggesting its exhibit similar function in regulation carotenoid biosynthesis. R2R3-MYB is a subfamily of MYB TF that plays a key role in diverse biological processes, especially in regulation of carotenoid and flavonoid biosynthesis [26]. RCP1, a R2R3-MYB member in *Mimulus lewisii*, is the first transcription factor that positively regulates carotenoid biosynthesis during flower development [54]. In tobacco, over-expression of an *LcMYB1* gene can induce anthocyanin accumulation in all tissues of transgenic line [55]. We further detect c42659_g1 and c61265_g1 are respectively homologous to *RCP1* and *LcMYB1* (Fig 5B), indicating the putative role in pigment biosynthesis regulation. As one of largest TF family in eukaryote, bHLH together with R2R3-MYB and WD40, construct a MYB-bHLH-WD40 transcription factors complex that actives the promoter of flavonoid biosynthesis related genes [56]. Xiang et al report CmbHLH2 binds the promoter of *CmDFR* (Dihydroflavonol 4-reductase, a key enzyme for flavonoid biosynthesis) and triggers the accumulation of anthocyanin [57]. TT8 is a key regulator controlling the regulation of flavonoid biosynthesis [58]. In *Medicago truncatula*, MtTT8 positively regulates a subset of genes involved in proanthocyanidin and anthocyanin [59]. In the current here, c80794_g1 is homologous to *TT8* and *CmbHLH2*, and three unigenes (c82524_g1, c82171_g1 and c19158_g1) together with *MtTT8* are grouped into a subgroup by phylogenetic analysis (Fig 5C), suggesting they may play a crucial role in regulation of flavonoid biosynthesis in orchid.

## Conclusions

We systematically compared the biochemical, cytological and transcriptome differences between YL and GL. Thousands of unigenes with differential expression were identified, and some of them were further validated by qRT-PCR. The activation of chlorophyll degeneration and flavonoid biosynthesis as well as repression of carotenoid biosynthesis are beneficial to the yellowing rhizome and leaves of YL. Our study provides further insights into the differences of the molecular mechanisms between two orchid cultivars and may help in future orchid breeding.

## Supporting information

S1 File Length distribution of assembled unigenes. (TIF)

S2 File DGEs identification and verification. (A) Identification of DGEs. The red and blue dots represented unigenes with up-regulation and down-regulation. (B) Validation of 10 randomly selected DEGs derived from RNA-seq using qRT-PCR. Error bars for qRT-PCR showed the standard deviation of three replicates.
(TIF)

S3 File Functional annotation of DEGs based on KEGG categorization. Significantly enriched biochemical pathways revealed by KEGG analysis. Bar showed enrichment factor. *, **, *** represented 0.05, 0.01 and 0.001 enrichment factor, respectively.
(TIF)

S1 Table Primers used in this paper. (DOCX)

S2 Table Gene accession numbers of other species used in this paper. (DOCX)

S3 Table Summary of YL and GL transcriptome sequencing data. (DOCX)

S4 Table Blast analysis of pigment-related unigenes in contrast to *C*. *ensifolium* and *C*. *sinense* databases in Orchistra2.0. (DOC)

S5 Table Nucleotide sequences of unigenes validated by qRT-PCR. (DOCX)
