# Supplementary figures and images for: Comparative transcriptome analysis provides global insight into gene expression differences between two orchid cultivars
Source: PLoS One. 2018 Jul 5;13(7):e0200155. doi: 10.1371/journal.pone.0200155 (PMC6033423; doi:10.1371/journal.pone.0200155)

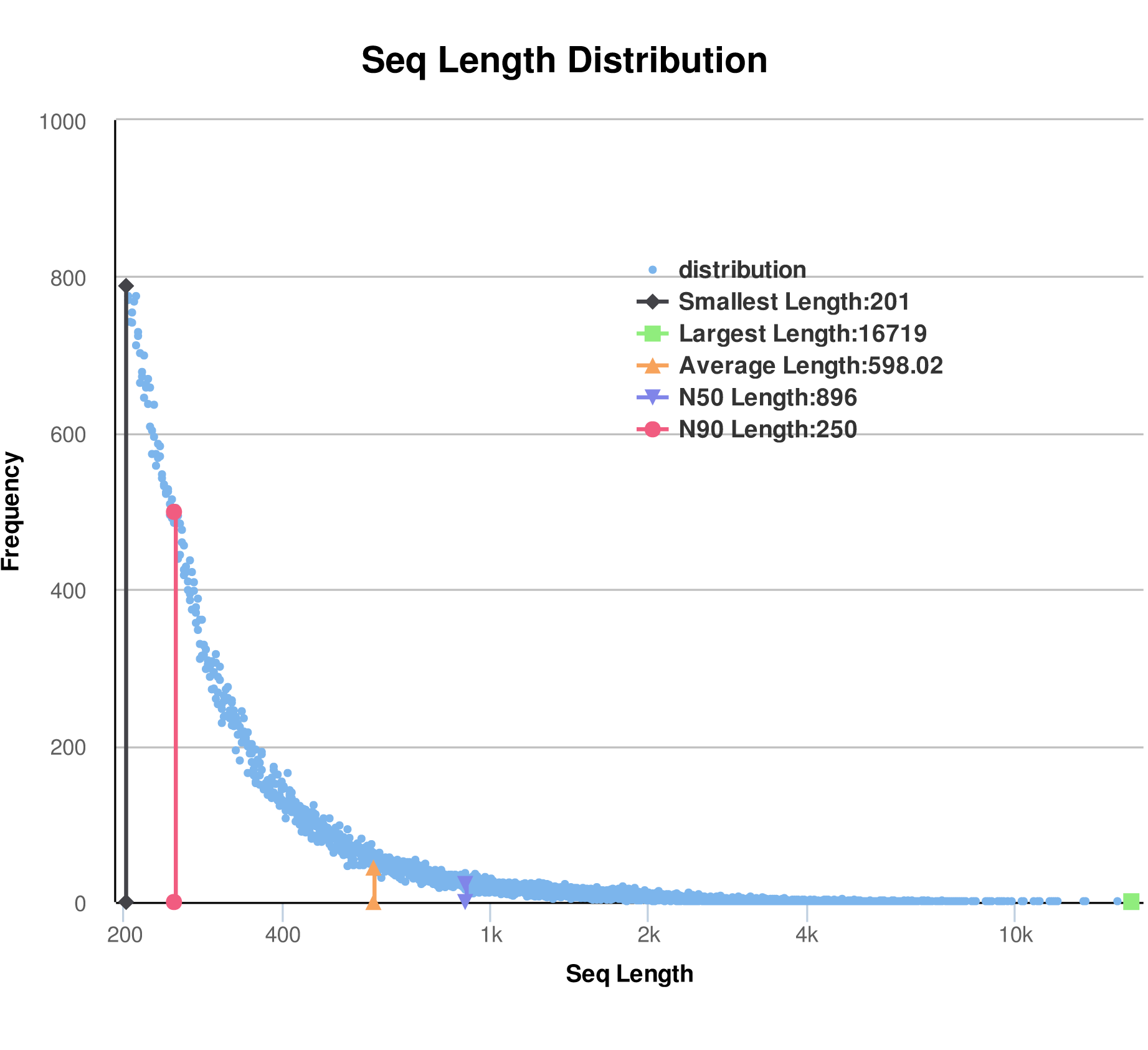

Supplement: S1 File — (TIF) [file pone.0200155.s001.tif]

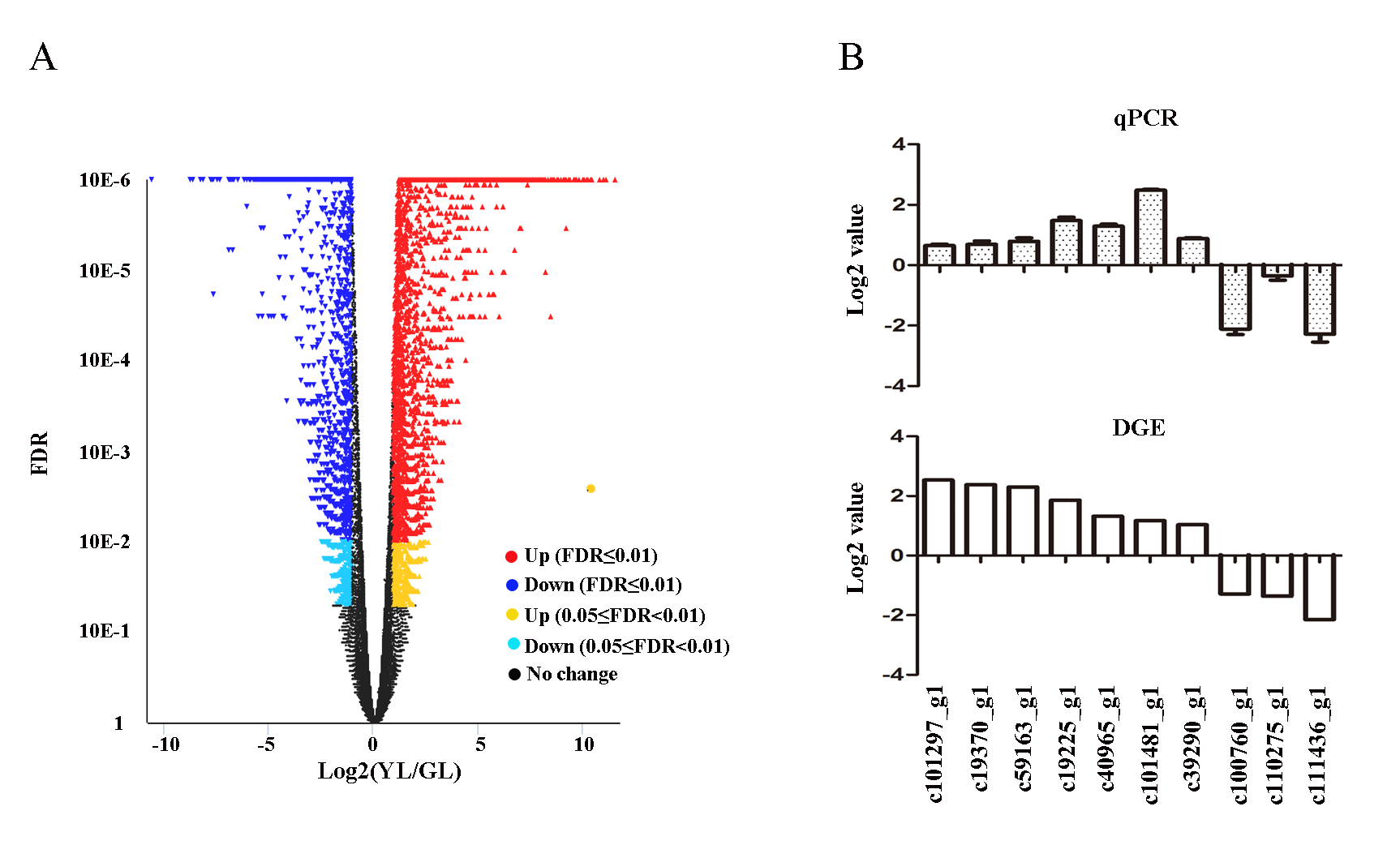

Supplement: S2 File — (A) Identification of DGEs. The red and blue dots represented unigenes with up-regulation and down-regulation. (B) Validation of 10 randomly selected DEGs derived from RNA-seq using qRT-PCR. Error bars for qRT-PCR showed the standard deviation of three replicates. (TIF) [file pone.0200155.s002.tif]

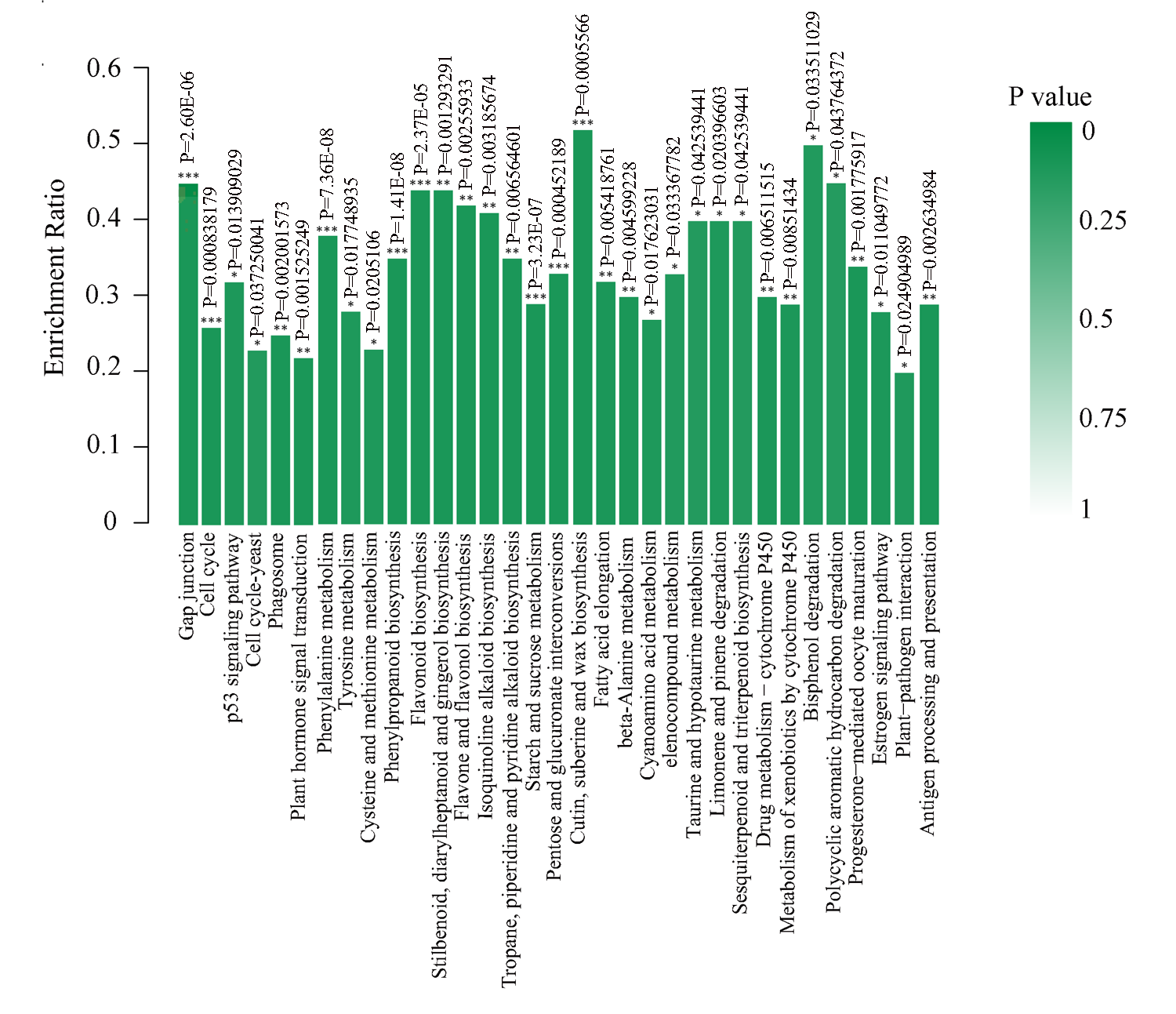

Supplement: S3 File — Significantly enriched biochemical pathways revealed by KEGG analysis. Bar showed enrichment factor. *, **, *** represented 0.05, 0.01 and 0.001 enrichment factor, respectively. (TIF) [file pone.0200155.s003.tif]
