# Supplementary material for: Comparative transcriptome analysis provides global insight into gene expression differences between two orchid cultivars
Source: PLoS One. 2018 Jul 5;13(7):e0200155. doi: 10.1371/journal.pone.0200155 (PMC6033423; doi:10.1371/journal.pone.0200155)
Supplement: S1 Table — (DOCX) [file pone.0200155.s004.docx]

**Table S1. Primers used in this paper.**

| Gene Name | | Primer sequence | Annotation | Amplification  Efficiency |
| --- | --- | --- | --- | --- |
| c101297_g1 | Forward: CGCTGTCCAGCTCCCC  Reverse: CGCTCAATCGCCCAAA | | Putative uncharacterized protein | 91.2% |
| c19370_g1 | Forward: TTTGGAAGACGAACAA  Reverse: CTGGAGAAGGAGGTGC | | Magnesium protoporphyrin IX methyltransferase | 87.9% |
| c59163_g1 | Forward: TGGTGCTGGCAAGATG  Reverse: ACGCAGTGCTGGTGAA | | Glutamyl-tRNA reductase 2-like isoform X2 | 85.3% |
| c19225_g1 | Forward: TCGTCTTCATCCGCCA  Reverse: CCCAACCATAATCCCC | | Chlorophyll a-b binding protein 13 | 94.6% |
| c40965_g1 | Forward: GGGTGGGTTGGATTAT  Reverse: TTGCCTGGGTAGAGTG | | Chlorophyll a/b-binding protein | 96.8% |
| c101481_g1 | Forward: CTACTGAGGCTGGCTT  Reverse: GGATTGTCGCTGGAAA | | RuBisCO large subunit-binding protein subunit beta | 89.2% |
| c39290_g1 | Forward: AACGATCCAGACCAGG  Reverse: TCTCTCAGCAGTGCCA | | Chlorophyll a-b binding protein CP26 | 90.7% |
| c100760_g1 | Forward: CTAATAACGACCCAAT  Reverse: GCAAAGAACACACAAA | | Pleiotropic drug resistance protein 12-like | 91.9% |
| c110275_g1 | Forward: GGTCTTACCACCATCA  Reverse: GGGAACCTTTTCTCTA | | Photosystem II 10 kDa phosphoprotein | 89.8% |
| c111436_g1 | Forward: CATCCATTGCTTGTCG  Reverse: ATCATTCTGCTCTGCC | | Alcohol dehydrogenase 1 | 93.4% |
| c4635_g1 | Forward: CATTGCGTTCATCGTT  Reverse: CAAGCCCCTCTTTTTT | | Uroporphyrinogen III synthase | 83.5% |
| c78740_g1 | Forward: TTCACTGGTTACTCTC  Reverse: AACTCAATCTTGTTCT | | O-methyltransferase | 86.8% |
| c52282_g1 | Forward: CCTCACGACCTTTCAA  Reverse: CTTTCCCCACTCTCTG | | flavonol synthase | 90.7% |
| c48794_g1 | Forward: GTTGCTCGGCTGTTCG  Reverse: AGTTGATGGTTTGGGA | | STAY-GREEN | 92.3% |
| c16388_g1 | Forward: TTGTAGCAACCAAGGC  Reverse: ACGGAAAAGAAAATCT | | Chalcone synthase | 91.6% |
| c7212_g1 | Forward: TTGGGGTGGGAGATGT  Reverse: CTGGGAGAGTGGAAGC | | zeaxanthin epoxidase | 88.8% |
| c4492_g1 | Forward: TTTTTACGGCGGGAAC  Reverse: GATGGAGGCGGAAGGT | | flavonoid 3'-hydroxylase | 91.3% |
| c42659_g1 | Forward: CCCTGTAGTTTCCGTT  Reverse: CCTTTCCACCTTGATT | | R2R3-MYB transcription factor | 86.5% |
| c51607_g1 | Forward: AGGATAAGTGAAAGGC  Reverse: GAGAAATCTCAGGTGC | | Transcription factor bHLH76-like | 92.7% |
| c82816_g1 | Forward: TTTTTAGTCATCCCTG  Reverse: GCTCTTATTTCTTTCG | | Transcription factor bHLH78-like | 84.2% |
| c80144_g1 | Forward: TTTCTCTCCATTTCTA  Reverse: CACATCTACTTGCCTC | | NAC transcription factor 25-like | 87.8% |
| ACTIN | Forward: AATCCCAAGGCAAACAGA  Reverse: CCATACCAGAATCCAG | | Actin | 92.8% |
