# Supplementary material for: Comparative transcriptome analysis provides global insight into gene expression differences between two orchid cultivars
Source: PLoS One. 2018 Jul 5;13(7):e0200155. doi: 10.1371/journal.pone.0200155 (PMC6033423; doi:10.1371/journal.pone.0200155)
Supplement: S2 Table — (DOCX) [file pone.0200155.s005.docx]

**Tabel S2. Gene accession numbers of other species used in this paper**

|  | Gene Name | Locus ID |
| --- | --- | --- |
| NACs | | |
|  | ATNAC001 | AT1G01010 |
|  | ATNAC002 | AT1G01720 |
|  | ATNAC003 | AT1G02220 |
|  | ATNAC004 | AT1G02230 |
|  | ATNAC005 | AT1G02250 |
|  | ATNAC006 | AT1G03490 |
|  | ATNAC007 | AT1G12260 |
|  | ATNAC008 | AT1G25580 |
|  | ATNAC009 | AT1G26870 |
|  | ATNAC010 | AT1G28470 |
|  | ATNAC011 | AT1G32510 |
|  | ATNAC012 | AT1G32770 |
|  | ATNAC013 | AT1G32870 |
|  | ATNAC014 | AT1G33060 |
|  | ATNAC015 | AT1G33280 |
|  | ATNAC016 | AT1G34180 |
|  | ATNAC017 | AT1G34190 |
|  | ATNAC018 | AT1G52880 |
|  | ATNAC019 | AT1G52890 |
|  | ATNAC020 | AT1G54330 |
|  | ATNAC021 | AT1G56010 |
|  | ATNAC022 | AT1G56010.2 |
|  | ATNAC023 | AT1G60280 |
|  | ATNAC024 | AT1G60350 |
|  | ATNAC025 | AT1G61110 |
|  | ATNAC026 | AT1G62700 |
|  | ATNAC027 | AT1G64105 |
|  | ATNAC028 | AT1G65910 |
|  | ATNAC029 | AT1G69490 |
|  | ATNAC030 | AT1G71930 |
|  | ATNAC031 | AT1G76420 |
|  | ATNAC032 | AT1G77450 |
|  | ATNAC033 | AT1G79580 |
|  | ATNAC034 | AT2G02450 |
|  | ATNAC035 | AT2G02450.2 |
|  | ATNAC036 | AT2G17040 |
|  | ATNAC037 | AT2G18060 |
|  | ATNAC038 | AT2G24430 |
|  | ATNAC039 | AT2G24430.2 |
|  | ATNAC040 | AT2G27300 |
|  | ATNAC041 | AT2G33480 |
|  | ATNAC042 | AT2G43000 |
|  | ATNAC043 | AT2G46770 |
|  | ATNAC044 | AT3G01600 |
|  | ATNAC045 | AT3G03200 |
|  | ATNAC046 | AT3G04060 |
|  | ATNAC047 | AT3G04070 |
|  | ATNAC048 | AT3G04420 |
|  | ATNAC049 | AT3G04430 |
|  | ATNAC050 | AT3G10480 |
|  | ATNAC051 | AT3G10490 |
|  | ATNAC052 | AT3G10490.2 |
|  | ATNAC053 | AT3G10500 |
|  | ATNAC054 | AT3G15170 |
|  | ATNAC055 | AT3G15500 |
|  | ATNAC056 | AT3G15510 |
|  | ATNAC057 | AT3G17730 |
|  | ATNAC058 | AT3G18400 |
|  | ATNAC059 | AT3G29035 |
|  | ATNAC060 | AT3G44290 |
|  | ATNAC061 | AT3G44350 |
|  | ATNAC062 | AT3G49530 |
|  | ATNAC063 | AT3G55210 |
|  | ATNAC064 | AT3G56530 |
|  | ATNAC065 | AT3G56560 |
|  | ATNAC066 | AT3G61910 |
|  | ATNAC067 | AT4G01520 |
|  | ATNAC068 | AT4G01540 |
|  | ATNAC069 | AT4G01550 |
|  | ATNAC070 | AT4G10350 |
|  | ATNAC071 | AT4G17980 |
|  | ATNAC072 | AT4G27410.2 |
|  | ATNAC073 | AT4G28500 |
|  | ATNAC074 | AT4G28530 |
|  | ATNAC075 | AT4G29230 |
|  | ATNAC076 | AT4G36160 |
|  | ATNAC077 | AT5G04400 |
|  | ATNAC078 | AT5G04410 |
|  | ATNAC079 | AT5G07680 |
|  | ATNAC080 | AT5G07680.2 |
|  | ATNAC081 | AT5G08790 |
|  | ATNAC082 | AT5G09330 |
|  | ATNAC083 | AT5G13180 |
|  | ATNAC084 | AT5G14000 |
|  | ATNAC085 | AT5G14490 |
|  | ATNAC086 | AT5G17260 |
|  | ATNAC087 | AT5G18270 |
|  | ATNAC088 | AT5G18300 |
|  | ATNAC089 | AT5G22290 |
|  | ATNAC090 | AT5G22380 |
|  | ATNAC091 | AT5G24590.2 |
|  | ATNAC092 | AT5G39610 |
|  | ATNAC093 | AT5G39690 |
|  | ATNAC094 | AT5G39820 |
|  | ATNAC095 | AT5G41090 |
|  | ATNAC096 | AT5G46590 |
|  | ATNAC097 | AT5G50820 |
|  | ATNAC098 | AT5G53950 |
|  | ATNAC099 | AT5G56620 |
|  | ATNAC100 | AT5G61430 |
|  | ATTNAC101 | AT5G62380 |
|  | ATNAC102 | AT5G63790 |
|  | ATNAC103 | AT5G64060 |
|  | ATNAC104 | AT5G64530 |
|  | ATNAC105 | AT5G66300 |
|  | CpNAC1 | KT364871.1 |
|  | CpNAC2 | KT372241.1 |
|  | SlNAC1 | NM_001247553.3 |
|  | SlNAC4 | NM_001278979.1 |
| R2R3-ATMYBs | | |
|  | ATMYB0 | [AT2G02450](http://www.arabidopsis.org/servlets/TairObject?id=33983&type=locus) |
|  | ATMYB1 | [AT3G09230](http://www.arabidopsis.org/servlets/TairObject?id=37199&type=locus) |
|  | ATMYB2 | [AT2G47190](http://www.arabidopsis.org/servlets/TairObject?id=35398&type=locus) |
|  | ATMYB3 | [AT1G22640](http://www.arabidopsis.org/servlets/TairObject?id=27053&type=locus) |
|  | ATMYB4 | [AT4G38620](http://www.arabidopsis.org/servlets/TairObject?id=127483&type=locus) |
|  | ATMYB5 | [AT3G13540](http://www.arabidopsis.org/servlets/TairObject?id=38860&type=locus) |
|  | ATMYB6 | [AT4G09460](http://www.arabidopsis.org/servlets/TairObject?id=129298&type=locus) |
|  | ATMYB7 | [AT2G16720](http://www.arabidopsis.org/servlets/TairObject?id=34542&type=locus) |
|  | ATMYB8 | AT1G35515 |
|  | ATMYB9 | [AT5G16770](http://www.arabidopsis.org/servlets/TairObject?id=131235&type=locus) |
|  | ATMYB10 | [AT3G12820](http://www.arabidopsis.org/servlets/TairObject?id=37946&type=locus) |
|  | ATMYB11 | [AT3G62610](http://www.arabidopsis.org/servlets/TairObject?id=36884&type=locus) |
|  | ATMYB12 | AT2G47460 |
|  | ATMYB13 | [AT1G06180](http://www.arabidopsis.org/servlets/TairObject?id=31226&type=locus) |
|  | ATMYB14 | AT2G31180 |
|  | ATMYB15 | AT3G23250 |
|  | ATMYB16 | AT5G15310 |
|  | ATMYB17 | AT3G61250 |
|  | ATMYB18 | AT4G25560 |
|  | ATMYB19 | [AT5G52260](http://www.arabidopsis.org/servlets/TairObject?id=130737&type=locus) |
|  | ATMYB20 | [AT1G66230](http://www.arabidopsis.org/servlets/TairObject?id=137715&type=locus) |
|  | ATMYB21 | [AT3G27810](http://www.arabidopsis.org/servlets/TairObject?id=38209&type=locus) |
|  | ATMYB22 | [AT5G40430](http://www.arabidopsis.org/servlets/TairObject?id=134147&type=locus) |
|  | ATMYB23 | [AT5G40330](http://www.arabidopsis.org/servlets/TairObject?id=134144&type=locus) |
|  | ATMYB24 | [AT5G40350](http://www.arabidopsis.org/servlets/TairObject?id=134148&type=locus) |
|  | ATMYB25 | [AT2G39880](http://www.arabidopsis.org/servlets/TairObject?id=34769&type=locus) |
|  | ATMYB26 | [AT3G13890](http://www.arabidopsis.org/servlets/TairObject?id=38035&type=locus) |
|  | ATMYB27 | [AT3G53200](http://www.arabidopsis.org/servlets/TairObject?id=40425&type=locus) |
|  | ATMYB28 | [AT5G61420](http://www.arabidopsis.org/servlets/TairObject?id=133203&type=locus) |
|  | ATMYB29 | [AT5G07690](http://www.arabidopsis.org/servlets/TairObject?id=132783&type=locus) |
|  | ATMYB30 | [AT3G28910](http://www.arabidopsis.org/servlets/TairObject?id=38495&type=locus) |
|  | ATMYB31 | [AT1G74650](http://www.arabidopsis.org/servlets/TairObject?id=28509&type=locus) |
|  | ATMYB32 | [AT4G34990](http://www.arabidopsis.org/servlets/TairObject?id=129008&type=locus) |
|  | ATMYB33 | [AT5G06100](http://www.arabidopsis.org/servlets/TairObject?id=131748&type=locus) |
|  | ATMYB34 | [AT5G60890](http://www.arabidopsis.org/servlets/TairObject?id=134566&type=locus) |
|  | ATMYB35 | [AT3G28470](http://www.arabidopsis.org/servlets/TairObject?id=38172&type=locus) |
|  | ATMYB36 | [AT5G57620](http://www.arabidopsis.org/servlets/TairObject?id=134711&type=locus) |
|  | ATMYB37 | [AT5G23000](http://www.arabidopsis.org/servlets/TairObject?id=135609&type=locus) |
|  | ATMYB38 | AT2G36890 |
|  | ATMYB39 | AT4G17785 |
|  | ATMYB40 | [AT5G14340](http://www.arabidopsis.org/servlets/TairObject?id=130794&type=locus) |
|  | ATMYB41 | [AT4G28110](http://www.arabidopsis.org/servlets/TairObject?id=129203&type=locus) |
|  | ATMYB42 | [AT4G12350](http://www.arabidopsis.org/servlets/TairObject?id=129995&type=locus) |
|  | ATMYB43 | [AT5G16600](http://www.arabidopsis.org/servlets/TairObject?id=134656&type=locus) |
|  | ATMYB44 | [AT5G67300](http://www.arabidopsis.org/servlets/TairObject?id=132479&type=locus) |
|  | ATMYB45 | [AT3G48920](http://www.arabidopsis.org/servlets/TairObject?id=40292&type=locus) |
|  | ATMYB46 | [AT5G12870](http://www.arabidopsis.org/servlets/TairObject?id=135766&type=locus) |
|  | ATMYB47 | [AT1G18710](http://www.arabidopsis.org/servlets/TairObject?id=30679&type=locus) |
|  | ATMYB48 | [AT3G46130](http://www.arabidopsis.org/servlets/TairObject?id=35752&type=locus) |
|  | ATMYB49 | [AT5G54230](http://www.arabidopsis.org/servlets/TairObject?id=133124&type=locus) |
|  | ATMYB50 | [AT1G57560](http://www.arabidopsis.org/servlets/TairObject?id=29511&type=locus) |
|  | ATMYB51 | [AT1G18570](http://www.arabidopsis.org/servlets/TairObject?id=29505&type=locus) |
|  | ATMYB52 | [AT1G17950](http://www.arabidopsis.org/servlets/TairObject?id=30008&type=locus) |
|  | ATMYB53 | [AT5G65230](http://www.arabidopsis.org/servlets/TairObject?id=134332&type=locus) |
|  | ATMYB54 | [AT1G73410](http://www.arabidopsis.org/servlets/TairObject?id=137933&type=locus) |
|  | ATMYB55 | [AT4G01680](http://www.arabidopsis.org/servlets/TairObject?id=129266&type=locus) |
|  | ATMYB56 | [AT5G17800](http://www.arabidopsis.org/servlets/TairObject?id=134908&type=locus) |
|  | ATMYB57 | [AT3G01530](http://www.arabidopsis.org/servlets/TairObject?id=37324&type=locus) |
|  | ATMYB58 | [AT1G16490](http://www.arabidopsis.org/servlets/TairObject?id=30345&type=locus) |
|  | ATMYB59 | [AT5G59780](http://www.arabidopsis.org/servlets/TairObject?id=134674&type=locus) |
|  | ATMYB60 | [AT1G08810](http://www.arabidopsis.org/servlets/TairObject?id=29159&type=locus) |
|  | ATMYB61 | [AT1G09540](http://www.arabidopsis.org/servlets/TairObject?id=27560&type=locus) |
|  | ATMYB62 | [AT1G68320](http://www.arabidopsis.org/servlets/TairObject?id=136910&type=locus) |
|  | ATMYB63 | [AT1G79180](http://www.arabidopsis.org/servlets/TairObject?id=137995&type=locus) |
|  | ATMYB64 | [AT5G11050](http://www.arabidopsis.org/servlets/TairObject?id=136101&type=locus) |
|  | ATMYB65 | [AT3G11440](http://www.arabidopsis.org/servlets/TairObject?id=36693&type=locus) |
|  | ATMYB66 | [AT5G14750](http://www.arabidopsis.org/servlets/TairObject?id=136167&type=locus) |
|  | ATMYB67 | [AT3G12720](http://www.arabidopsis.org/servlets/TairObject?id=37953&type=locus) |
|  | ATMYB68 | [AT5G65790](http://www.arabidopsis.org/servlets/TairObject?id=134069&type=locus) |
|  | ATMYB69 | [AT4G33450](http://www.arabidopsis.org/servlets/TairObject?id=127190&type=locus) |
|  | ATMYB70 | [AT2G23290](http://www.arabidopsis.org/servlets/TairObject?id=34345&type=locus) |
|  | ATMYB71 | [AT3G24310](http://www.arabidopsis.org/servlets/TairObject?id=37856&type=locus) |
|  | ATMYB72 | [AT1G56160](http://www.arabidopsis.org/servlets/TairObject?id=27446&type=locus) |
|  | ATMYB73 | [AT4G37260](http://www.arabidopsis.org/servlets/TairObject?id=126620&type=locus) |
|  | ATMYB74 | [AT4G05100](http://www.arabidopsis.org/servlets/TairObject?id=126712&type=locus) |
|  | ATMYB75 | [AT1G56650](http://www.arabidopsis.org/servlets/TairObject?id=29514&type=locus) |
|  | ATMYB76 | [AT5G07700](http://www.arabidopsis.org/servlets/TairObject?id=132785&type=locus) |
|  | ATMYB77 | AT3G50060 |
|  | ATMYB78 | [AT5G49620](http://www.arabidopsis.org/servlets/TairObject?id=132433&type=locus) |
|  | ATMYB79 | [AT4G13480](http://www.arabidopsis.org/servlets/TairObject?id=130215&type=locus) |
|  | ATMYB80 | [AT5G56110](http://www.arabidopsis.org/servlets/TairObject?id=133010&type=locus) |
|  | ATMYB81 | [AT2G26960](http://www.arabidopsis.org/servlets/TairObject?id=34459&type=locus) |
|  | ATMYB82 | [AT5G52600](http://www.arabidopsis.org/servlets/TairObject?id=500229962&type=locus) |
|  | ATMYB83 | [AT3G08500](http://www.arabidopsis.org/servlets/TairObject?id=40674&type=locus) |
|  | ATMYB84 | [AT3G49690](http://www.arabidopsis.org/servlets/TairObject?id=39610&type=locus) |
|  | ATMYB85 | [AT4G22680](http://www.arabidopsis.org/servlets/TairObject?id=129155&type=locus) |
|  | ATMYB86 | [AT5G26660](http://www.arabidopsis.org/servlets/TairObject?id=130953&type=locus) |
|  | ATMYB87 | [AT4G37780](http://www.arabidopsis.org/servlets/TairObject?id=129811&type=locus) |
|  | ATMYB88 | [AT2G02820](http://www.arabidopsis.org/servlets/TairObject?id=34377&type=locus) |
|  | ATMYB89 | [AT5G39700](http://www.arabidopsis.org/servlets/TairObject?id=133422&type=locus) |
|  | ATMYB90 | [AT1G66390](http://www.arabidopsis.org/servlets/TairObject?id=137239&type=locus) |
|  | ATMYB91 | [AT2G37630](http://www.arabidopsis.org/servlets/TairObject?id=31595&type=locus) |
|  | ATMYB92 | [AT5G10280](http://www.arabidopsis.org/servlets/TairObject?id=130762&type=locus) |
|  | ATMYB93 | [AT1G34670](http://www.arabidopsis.org/servlets/TairObject?id=28863&type=locus) |
|  | ATMYB94 | [AT3G47600](http://www.arabidopsis.org/servlets/TairObject?id=36445&type=locus) |
|  | ATMYB95 | [AT1G74430](http://www.arabidopsis.org/servlets/TairObject?id=28497&type=locus) |
|  | ATMYB96 | [AT5G62470](http://www.arabidopsis.org/servlets/TairObject?id=131919&type=locus) |
|  | ATMYB97 | [AT4G26930](http://www.arabidopsis.org/servlets/TairObject?id=126810&type=locus) |
|  | ATMYB98 | [AT4G18770](http://www.arabidopsis.org/servlets/TairObject?id=127904&type=locus) |
|  | ATMYB99 | [AT5G62320](http://www.arabidopsis.org/servlets/TairObject?id=133794&type=locus) |
|  | ATMYB100 | AT2G25230 |
|  | ATMYB101 | [AT2G32460](http://www.arabidopsis.org/servlets/TairObject?id=34603&type=locus) |
|  | ATMYB102 | AT4G21440 |
|  | ATMYB103 | AT1G63910 |
|  | ATMYB104 | [AT2G26950](http://www.arabidopsis.org/servlets/TairObject?id=31392&type=locus) |
|  | ATMYB105 | [AT1G69560](http://www.arabidopsis.org/servlets/TairObject?id=26671&type=locus) |
|  | ATMYB106 | AT3G01140 |
|  | ATMYB107 | AT3G02940 |
|  | ATMYB108 | AT3G06490 |
|  | ATMYB109 | [AT3G55730](http://www.arabidopsis.org/servlets/TairObject?id=36405&type=locus) |
|  | ATMYB110 | AT3G29020 |
|  | ATMYB111 | AT5G49330 |
|  | ATMYB112 | AT1G48000 |
|  | ATMYB113 | AT1G66370 |
|  | ATMYB114 | AT1G66380 |
|  | ATMYB115 | AT5G40360 |
|  | ATMYB116 | [AT1G25340](http://www.arabidopsis.org/servlets/TairObject?id=30367&type=locus) |
|  | ATMYB117 | AT1G26780 |
|  | ATMYB118 | [AT3G27785](http://www.arabidopsis.org/servlets/TairObject?id=227475&type=locus) |
|  | ATMYB119 | AT5G58850 |
|  | ATMYB120 | [AT5G55020](http://www.arabidopsis.org/servlets/TairObject?id=131643&type=locus) |
|  | ATMYB121 | AT3G30210 |
|  | ATMYB122 | AT1G74080 |
|  | ATMYB122(TT3) | AT5G35550 |
|  | ATMYB124 | AT1G14350 |
|  | EsMYB9 | JN426956.1 |
|  | HlMYB8 | HG983335 |
|  | GmMYBJ3 | KU664645 |
|  | CrMYB68 | KY612511.2 |
|  | LcMYB1 | KY302802.1 |
| bHLHs | | |
|  | ATbHLH001 | AT5G41315 |
|  | ATbHLH002 | AT1G63650 |
|  | ATbHLH003 | AT4G16430 |
|  | ATbHLH004 | AT4G17880 |
|  | ATbHLH005 | AT5G46760 |
|  | ATbHLH006 | AT1G32640 |
|  | ATbHLH007 | AT1G03040 |
|  | ATbHLH008 | AT1G09530 |
|  | ATbHLH009 | AT2G43010 |
|  | ATbHLH010 | AT2G31220 |
|  | ATbHLH011 | AT4G36060 |
|  | ATbHLH012 | AT4G00480 |
|  | ATbHLH013 | AT1G01260 |
|  | ATbHLH014 | AT4G00870 |
|  | ATbHLH015 | AT2G20180 |
|  | ATbHLH016 | AT4G00050 |
|  | ATbHLH017 | AT2G46510 |
|  | ATbHLH018 | AT2G22750 |
|  | ATbHLH019 | AT2G22760 |
|  | ATbHLH020 | AT2G22770 |
|  | ATbHLH021 | AT2G16910 |
|  | ATbHLH022 | AT4G21330 |
|  | ATbHLH023 | AT4G28790 |
|  | ATbHLH024 | AT4G36930 |
|  | ATbHLH025 | AT4G37850 |
|  | ATbHLH026 | AT1G02340 |
|  | ATbHLH027 | AT4G29930 |
|  | ATbHLH028 | AT5G46830 |
|  | ATbHLH029 | AT2G28160 |
|  | ATbHLH030 | AT1G68810 |
|  | ATbHLH031 | AT1G59640 |
|  | ATbHLH032 | AT3G25710 |
|  | ATbHLH033 | AT1G12860 |
|  | ATbHLH034 | AT3G23210 |
|  | ATbHLH035 | AT5G57150 |
|  | ATbHLH036 | AT5G51780 |
|  | ATbHLH037 | AT3G50330 |
|  | ATbHLH038 | AT3G56970 |
|  | ATbHLH039 | AT3G56980 |
|  | ATbHLH040 | AT4G00120 |
|  | ATbHLH041 | AT5G56960 |
|  | ATbHLH042 (TT8) | AT4G09820 |
|  | ATbHLH043 | AT5G09750 |
|  | ATbHLH044 | AT1G18400 |
|  | ATbHLH045 | AT3G06120 |
|  | ATbHLH046 | AT5G10570 |
|  | ATbHLH047 | AT3G47640 |
|  | ATbHLH048 | AT2G42300 |
|  | ATbHLH049 | AT1G68920 |
|  | ATbHLH050 | AT1G73830 |
|  | ATbHLH051 | AT2G40200 |
|  | ATbHLH052 | AT1G30670 |
|  | ATbHLH053 | AT2G34820 |
|  | ATbHLH054 | AT1G27740 |
|  | ATbHLH059 | AT4G02590 |
|  | ATbHLH060 | AT3G57800 |
|  | ATbHLH061 | AT5G10570 |
|  | ATbHLH062 | AT3G07340 |
|  | ATbHLH063 | AT4G34530 |
|  | ATbHLH064 | AT2G18300 |
|  | ATbHLH065 | AT3G59060 |
|  | ATbHLH066 | AT2G24260 |
|  | ATbHLH067 | AT3G61950 |
|  | ATbHLH068 | AT4G29100 |
|  | ATbHLH069 | AT4G30980 |
|  | ATbHLH070 | AT2G46810 |
|  | ATbHLH071 | AT5G46690 |
|  | ATbHLH072 | AT5G61270 |
|  | ATbHLH073 | AT5G67110 |
|  | ATbHLH074 | AT1G10120 |
|  | ATbHLH075 | AT1G25330 |
|  | ATbHLH076 | AT1G26260 |
|  | ATbHLH077 | AT3G23690 |
|  | ATbHLH078 | AT5G48560 |
|  | ATbHLH079 | AT5G62610 |
|  | ATbHLH080 | AT1G35460 |
|  | ATbHLH081 | AT4G09180 |
|  | ATbHLH082 | AT5G58010 |
|  | ATbHLH083 | AT1G66470 |
|  | ATbHLH084 | AT2G14760 |
|  | ATbHLH085 | AT4G33880 |
|  | ATbHLH086 | AT5G37800 |
|  | ATbHLH087 | AT3G21330 |
|  | ATbHLH088 | AT5G67060 |
|  | ATbHLH089 | AT1G06170 |
|  | ATbHLH090 | AT1G10610 |
|  | ATbHLH091 | AT2G31210 |
|  | ATbHLH092 | AT5G43650 |
|  | ATbHLH093 | AT5G65640 |
|  | ATbHLH094 | AT1G22490 |
|  | ATbHLH095 | AT1G49770 |
|  | ATbHLH096 | AT1G72210 |
|  | ATbHLH097 | AT3G24140 |
|  | ATbHLH098 | AT5G53210 |
|  | ATbHLH099 | AT5G65320 |
|  | ATbHLH102 | AT1G69010 |
|  | ATbHLH105 | AT5G54680 |
|  | ATbHLH106 | AT2G41130 |
|  | ATbHLH116 | AT3G26744 |
|  | ATbHLH100 | AT2G41240 |
|  | ATbHLH101 | AT5G04150 |
|  | ATbHLH103 | AT4G21340 |
|  | ATbHLH104 | AT4G14410 |
|  | ATbHLH107 | AT3G56770 |
|  | ATbHLH108 | AT1G25310 |
|  | ATbHLH109 | AT1G68240 |
|  | ATbHLH110 | AT1G27660 |
|  | ATbHLH111 | AT1G31050 |
|  | ATbHLH112 | AT1G61660 |
|  | ATbHLH113 | AT3G19500 |
|  | ATbHLH114 | AT4G05170 |
|  | ATbHLH115 | AT1G51070 |
|  | ATbHLH117 | AT3G22100 |
|  | ATbHLH118 | AT4G25400 |
|  | ATbHLH119 | AT4G28811 |
|  | ATbHLH120 | AT5G51790 |
|  | ATbHLH121 | AT3G19860 |
|  | ATbHLH122 | AT1G51140 |
|  | ATbHLH123 | AT3G20640 |
|  | ATbHLH124 | AT2G46970 |
|  | ATbHLH125 | AT1G62975 |
|  | ATbHLH126 | AT4G25410 |
|  | ATbHLH127 | AT4G28815 |
|  | ATbHLH128 | AT1G05805 |
|  | ATbHLH129 | AT2G43140 |
|  | ATbHLH130 | AT2G42280 |
|  | ATbHLH132 | AT3G62090 |
|  | ATbHLH133 | AT2G20095 |
|  | ATbHLH134 | AT5G15160 |
|  | ATbHLH135 | AT1G74500 |
|  | ATbHLH136 | AT5G39860 |
|  | ATbHLH137 | AT5G50915 |
|  | ATbHLH138 | AT2G31215 |
|  | ATbHLH139 | AT5G43175 |
|  | ATbHLH140 | AT5G01310 |
|  | ATbHLH141 | AT5G38860 |
|  | ATbHLH142 | AT5G64340 |
|  | ATbHLH143 | AT5G09460 |
|  | ATbHLH144 | AT1G29950 |
|  | ATbHLH145 | AT5G50010 |
|  | ATbHLH146 | AT4G30180 |
|  | ATbHLH147 | AT3G17100 |
|  | ATbHLH148 | AT3G06590 |
|  | ATbHLH149 | AT1G09250 |
|  | ATbHLH150 | AT3G05800 |
|  | ATbHLH151 | AT2G47270 |
|  | ATbHLH152 | AT1G22380 |
|  | ATbHLH153 | AT1G05710 |
|  | ATbHLH154 | AT2G31730 |
|  | ATbHLH155 | AT2G31280 |
|  | ATbHLH156 | AT2G27230 |
|  | ATbHLH157 | AT1G64625 |
|  | ATbHLH158 | AT2G43060 |
|  | ATbHLH159 | AT4G30410 |
|  | ATbHLH160 | AT1G71200 |
|  | ATbHLH161 | AT3G47710 |
|  | ATbHLH162 | AT4G20970 |
|  |  | AT1G10585 |
|  | MtTT8 | KM892777.1 |
|  | CmbHLH2 | KT724056.1 |
