# Supplementary material for: Comparative transcriptome analysis provides global insight into gene expression differences between two orchid cultivars
Source: PLoS One. 2018 Jul 5;13(7):e0200155. doi: 10.1371/journal.pone.0200155 (PMC6033423; doi:10.1371/journal.pone.0200155)
Supplement: S3 Table — (DOCX) [file pone.0200155.s006.docx]

Tabel S3 Summary of YL and GL transcriptome sequencing data

|  | GL | YL |
| --- | --- | --- |
| Number of clean reads | 39,557,830 | 38,536,724 |
| Number of base pairs | 5,685,015,511 | 5,503,245,825 |
| Q20 (%) | 98.01 | 97.89 |
| Q30 (%) | 93.41 | 93.10 |
