# Supplementary material for: Comparative transcriptome analysis provides global insight into gene expression differences between two orchid cultivars
Source: PLoS One. 2018 Jul 5;13(7):e0200155. doi: 10.1371/journal.pone.0200155 (PMC6033423; doi:10.1371/journal.pone.0200155)
Supplement: S4 Table — (DOC) [file pone.0200155.s007.doc]

**Table S4. Blast analysis of pigment-related unigenes in contrast to *C. ensifolium* and *C. sinense* databases in Orchistra2.0.**

| ***Cymbidium longibracteatum*** | | | ***Cymbidium ensifolium*** | | | | ***Cymbidium sinense*** | | | |  |
| --- | --- | --- | --- | --- | --- | --- | --- | --- | --- | --- | --- |
| **Gene ID** | **Length** | | **Gene ID** | **Length** | | **Total score** | **Gene ID** | **Length** | **Total score** | | **Annotation** |
| **NAC TF** | | | | | | | | | | | |
| **c42861_g1** | 1174 | CETC002221 | | 1330 | 0 | | CSTC025682 | 956 | 2.11×10^-43^ | NAC transcription factor 027 | |
| **c105949_g1** | 1457 | CETC006804 | | 1379 | 0 | | CSTC012746 | 876 | 0 | NAC transcription factor 29-like | |
| **c24765_g1** | 1243 | CETC008319 | | 1326 | 0 | | CSTC015390 | 340 | 4.90×10^-172^ | NAC domain-containing protein 94 | |
| **c80144_g1** | 1552 | CETC003107 | | 2278 | 0 | | CSTC006431 | 638 | 9.75×10^-138^ | NAC transcription factor 25-like | |
| **bHLH TF** | | | | | | | | | | | |
| **c51607_g1** | 911 | CETC009076 | | 2017 | 46.37 | | CSTC014019 | 472 | 68.01 | Transcription factor bhlh76-like | |
| **c79074_g1** | 1024 | CETC001868 | | 394 | 48.17 | | CSTC014019 | 472 | 66.21 | Transcription factor bhlh76-like | |
| **c82171_g1** | 2041 | CETC016146 | | 1187 | 1988.60 | | CSTC001894 | 594 | 933.63 | Transcription factor bhlh18-like | |
| **c82524_g1** | 921 | CETC027439 | | 1218 | 1625.61 | | CSTC016083 | 346 | 607.22 | Transcription factor bhlh93-like | |
| **c81897_g1** | 1035 | CETC029205 | | 1662 | 1801.05 | | CSTC021247 | 334 | 163.59 | Transcription factor bhlh96-like | |
| **c22345_g1** | 1501 | CETC022176 | | 1280 | 2217.63 | | CSTC010734 | 314 | 558.53 | Transcription factor bhlh71-like | |
| **c27046_g1** | 1145 | CETC025347 | | 1853 | 2004.83 | | CSTC020120 | 344 | 612.63 | Transcription factor bhlh62-like | |
| **c19158_g1** | 1283 | CETC005185 | | 1344 | 2264.51 | | CSTC010536 | 754 | 1290.18 | Transcription factor bhlh18-like | |
| **c40476_g1** | 1612 | CETC003063 | | 1570 | 2706.34 | | CSTC021247 | 334 | 201.46 | Transcription factor bhlh94-like | |
| **c58741_g1** | 2009 | CETC004541 | | 2599 | 3499.82 | | CSTC019775 | 706 | 613.91 | Transcription factor bhlh49-like isoform X1 | |
| **c82816_g1** | 1816 | CETC029761 | | 1854 | 3178.82 | | CSTC009477 | 555 | 976.39 | Transcription factor bhlh78-like | |
| **c80794_g1** | 948 | CETC016990 | | 1022 | 1562.49 | | CSTC010177 | 459 | 810.48 | Transcription factor bhlh47-like | |
| **R2R3-MYB TF** | | | | | | | | | | | |
| **c42659_g1** | 1122 | CETC026110 | | 2120 | 1970.57 | | CSTC008644 | 838 | 1476.44 | R2R3-MYB transcription factor | |
| **c61265_g1** | 1293 | CETC024333 | | 1272 | 2237.46 | | CSTC025165 | 1085 | 1903.32 | R2R3-myb transcription factor | |
| **Chlorophyll metabolism** | | | | | | | | | | | |
| **c4635_g1** | 1433 | CETC010849 | | 2905 | 904.26 | | CSTC011875 | 702 | 524.26 | Uroporphyrinogen decarboxylase 1 | |
| **c19370_g1** | 1516 | CETC015524 | | 1445 | 2475.51 | | CSTC018219 | 1354 | 2360.09 | Magnesium protoporphyrin IX methyltransferase | |
| **c48794_g1** | 982 | CETC007271 | | 1425 | 71.10 | | CSTC027346 | 340 | 614.43 | STAY-GREEN | |
| **Carotenoid biosynthesis** | | | | | | | | | | | |
| **c7212_g1** | 1706 | CETC023186 | | 987 | 1754.16 | | CSTC008069 | 401 | 719.03 | Zeaxanthin epoxidase | |
| **Flavonoids biosynthesis** | | | | | | | | | | | |
| **c16388_g1** | 250 | CETC014018 | | 1571 | 338.52 | | CSTC011562 | 317 | 159.98 | Chalcone synthase | |
| **c48963_g1** | 1000 | CETC014018 | | 1571 | 1424.14 | | CSTC008601 | 515 | 841.66 | Chalcone synthase 4 | |
| **c63571_g1** | 415 | CETC001356 | | 1771 | 690.17 | | CSTC025873 | 311 | 544.10 | Flavonoid 3' hydroxylase | |
| **c4492_g1** | 1225 | CETC001356 | | 1771 | 2100.41 | | CSTC001589 | 500 | 860.98 | Flavonoid 3' hydroxylase | |
| **c52282_g1** | 1208 | CETC029436 | | 1271 | 2091.39 | | CSTC003334 | 975 | 1718.09 | Flavonol synthase | |
| **c78740_g1** | 1105 | CETC008132 | | 1097 | 1887.61 | | CSTC016373 | 1030 | 1806.46 | O-methyltransferase | |
| **c4645_g1** | 1038 | CETC007893 | | 1211 | 1831.71 | | CSTC026718 | 614 | 1058.06 | O-methyltransferase-2 | |
