# Supplementary material for: Comparative transcriptome analysis provides global insight into gene expression differences between two orchid cultivars
Source: PLoS One. 2018 Jul 5;13(7):e0200155. doi: 10.1371/journal.pone.0200155 (PMC6033423; doi:10.1371/journal.pone.0200155)
Supplement: S5 Table — (DOCX) [file pone.0200155.s008.docx]

**Tabel S5. Nucleotide sequences of unigenes validated by qRT-PCR.**

>c48794_g1 [organism= *Cymbidium longibracteatum*] STAY-GREEN protein

GAAGGGAAAATAATATAAGATAAAAATAATACAGCTATTGATCATCATACCTCAATTTGAAGATCTCTGGCGTCCAACATGGGCTCCGCGGCGGTCCTGCTGCCTACTCCCACATTGCAGCATCGGTCTTCTTCTCCCTCCCGCCGCCTCTCTATCAATCGCCACCTCCTCCGTCCCGGTCGGCGGTCCGTTTTACCGGTTGCTCGGCTGTTCGGACCGGCGATCTTCGAGGCGTCTAAGCTGAAAGTTATGTTCCTTGGAGTGGACGACCGGAAGCATCCGGAGAAGCTACCGCGCGCCTATACTCTTACCCACAGCGACGTCACAGCCCGTCTAACCCTTGCCATCTCCCAAACCATCAACTGGGCACAGCTGCAGGGCTGGTATCACAAGCTGCAGAGAGACGAGGTGGTGGCCGAATGGAGGAAGGTTCGAGGAAAGATGTCGCTGCACGTCCACTGCCACATCAGCGGCGGCCACTTCCTCCTCGACCTCGTCGCCAACCTCCGCCACCACATCTTCTCGAAAGAACTTCCGGTGGTTCTCAAGGCCTTCGTCCACGGCGACGGTGGCCTCCTCAGGACTTACCCGGATCTCGAGGACGCCCTCGTCTGGGTCTATTTCCATTCCAACTCCCCCGACCTCCACAGCGTCGAGTGCTGGGGCCCGCTCCGCGAGGCGGCGGAGATCGGCCGGCCGGAAAAGCCGGCTTCAGATGGCGTCAGCGAGGTCGAGAAGAGCTGGCCCACGCCGCACCAGCGTTGCGCGACTGACTGCGACTGCTGCTTCCCGGCGCATAGCTTGATTCCGTGGCCAATCGCCGAGATGAAGTCCGGTCAGCCGCAATAGTAGTATAGCTTGAATCGGACACGCAACACGTTTATTATATGATTATGATCAGAAAATTTGATTGAACGCTTAGCATAGTATAGGATTGATTCTAAATTTGTACATAGAAAAATGTTTAAAGCATTGAACGTAC

>c19370_g1 [organism= *Cymbidium longibracteatum*] magnesium protoporphyrin IX methyltransferase

GTTTACATTTCTCAGATACTCTATTTATGTGTTCAATATTAGTATATTCACACTGTTGTATGCCAACAGCCTATCCATCAGTACACCAAGTTCCATTTGGAAGACGAACAATATCACCAACCTTATCCGAAATCCACCACTCACCCCTAAAGCTCACAAATCCGAAATCCATCACGAATTATATTTCCGTTTAACAAAATTAAAAAATTCCAAAAACCCATTAGAGTATCCAATGGCTTCTTTCGCCACCGTTCTATCCTCCGGCTCCCATGCCCGCACCTCCTTCTCCAGCCTCCCAACACCCACCCCCCGCCGCCGACCCGCCACCAAAACCCTCGCCTTGCCACCAGTCCCCCTCGCCGCCGACCTCTCCCTCGACTCCCCAGCCGCCCTAGCCGTTCTCGGCAGCGCTGCCCTCGCCGCCGCTGTCTCCCTCACCGACCCCGAACGCCGCCGCCAACAACAAGCCCAAGAGATCGGCGGCACCGACAAAGAAGTCGTTCGCGACTACTTCAACACCACCGGCTTCGACCGCTGGAAGAAGATCTACGGTGAGGCGACCGAAGGCGTTAACAAGGTCCAGCTCGACATTCGCATCGGCCACTCCAAGACCGTGGAAAACGCCATCCTCATGCTCAAGGACGGCGGACCCCTTGCCGGGGTTAGCGTCTGCGACGCAGGCTGTGGAACCGGAAACCTCGCGATCCCGCTCGCACTCGAGGGGGCGATCGTGACAGCCAACGATATCTCGGCGGCGATGGTGGCGGAGGCGCGGCAGCAGGCAGAGGAAGCGATTAGAGGCGTCGAGGGGGTGAGAATGCCGGAGTTTGAGGTCAAGGATTTGGAGGAGTTGGAGGGGAAGTATCATACGGTGGTATGTCTCGATGTGCTGATACATTACCCGCAGGAAAAGGCGGAAGTGATGATCGCGCATCTGGCTTCGCTTGCGGAGAAGAGATTGCTGCTTAGCTTTGCGCCAAAGACGACGTATTATAGTTTGTTGAAGAGGCTCGGCGAGCTATTTCCAGGGCCGTCGAAGGCGACGAGAGCGTATCTACATGCAGAGAGCGATGTGGAGCGGGCGCTTGAGAGGCTGGGGTGGCGCGTGAGGAAGAGGGGATTTATCTCTACGCAGTTCTACTTCGCCAAGCTCCTCGAGGCCGTCCCGGCCGCCTGATTCTGGGATGGCTCGTGATTTTGAAGAACTTATGATTCAATTAGGCTGAGTTAAGCAATTGGGAACTGTTCATGAATGATTTTATCTGATCTTCTTAGGCGAGATCCTTACTCCTATGTACTGAATTTTGTAACTTTTTGGTGTCTTCTTTAAAAGACAATTCGGAGTTTTCGGAGGGTTGCTTGTGCTTCCCTGAAAATTAGGGCATGTTTGGTTGTGGGAGAGCAGCTGAATCGCTTGTGTTCTACTTGTTACTTTGGGCAGTAGGGCAATGTGATTGGAGTTTACTTGTTAGGAGTCGTGCTTCGCTATTTGAAAATAAATAAATAATTATTTGAT

>c4635_g1 [organism= *Cymbidium longibracteatum*] uroporphyrinogen III synthase

GTGTGCAGAACATTTTCTGCTTTCAGGTCTCTACGAGGAATAGGTGAAGACAGTGAATGCATGGCAAAAACTGCATTGCAGACATCTCTTAAAATTGAAAGAACCTTCTCCTCTAAGTATGCTATTCTCCTGCTTTCAAGAGCATTAACCAAAGACTCCCTGCAGAATTCTAACACAAGAAGCATCTCCTTCCTTCGACCCATGTCTAATATACTATGTGCAACCAAAGTAACAACATTTGGATGCCCTTTAAGCAGTTTCATCACTTGGCTCTCCTTTATCACTAGATCAAGTGACTCCCCATCTTGGCATATCATGAGCTTAAGAGCATATTATTTTGATACATTTGCAGCATCATGAGCAAGATACACACAAGAAAAGCCTCCTTCAGCAATCATGTTCTATACACGAAGCTCTTCACTGCCAATTTCAATTAGTTTGCCTTCTAATCCAGATTTTTCTTTCCCAGAGAAAGGTTTGAACATCCACATGTCTTAAGAGATCCGAAAATTGTAGAAACGAAGCAAAGGCAAAGATTGCCAATGATCAATCCCGTGACAGTCATCGTGGCCACACTGGCGTGGGGAAGCGAGAAATGAGAGCCTTTTTCTTCCTTCCTCTGCCGAATGAACACCAAATGCAGGTAGAGGGGTAAGTATGTCAAAGAAAATTATAACACCATCTAGATGGAAAGCTCTCCAAGGCTGCAAAGAAGTTTCCACAATGAGGTCAACTGTCTCTGACCTCACTCTGAAGGACGGATGTCTCTGCAAGTTTTCTATAGATAGCCATGTACCTTCCTGCTTGGCGCATCATCCATGCTGGAGGCCTGCTAACAAGATTCTGCCTAGCAGCTTGAGCCAATAGTGGATCCGAAGAAGAGCTTACCCTAGTAAATCTCTCAGAGTTTTTTTTGTTTTTTTCTGAGGAAGGAAGCCCTACTTCTTTCTGCGGGAGGAAGCACTACTTCGCACTATAACACCACTAGCGACCCAGGAATGATTCGGATGGGCTGCAACTTGTAGACCTACTTCTAGGCGGCACCGAAAATATTTGCATTGCGTTCATCGTTGAATTCTCTTCTTCAGTTCACAGTGGGAGGTAGCCTTCTTCTTCTCCTGTGCTTCCTTATCAGATACATGTGTGCCTTCGGCTGCTTTTCGTGGAGAGGGTAAGGAGGGCGAGAGAGAAGAAAGAGAGCCAACCGTTTTTGCGGTTCCTCAACCGAACGAAGAAAAAAGAGGGGCTTGCATGCTTTGTCGTTTTAAGGAGGATGTGCGTGCGGTTGAGCGAACGAACAAGAGAGAGGGAACGGTTGCTCGGCTTGAGATTGAGCGAAATTGAACGAGGGGGGGAAGAGATGCGAGATCATGTGCTATCCAGCTCAAGTCGACCGTGAATAGGAGATTTAGGTTGGTTTGGGTTATTAGGGA

>c7212_g1 [organism= *Cymbidium longibracteatum*] zeaxanthin epoxidase

GAGCTTTTGACGCATTTTTGACCATTTGTTTCCCTTCAGTCTTGAGAGAATTGTGGCTTTAACCAATTCGGCGGCACCTTCCACGTTCTATTCATTCGCTAAAATCCATCAGCTGCTCTTCAATTTGTTTCGTCGTTTCGCTCCTCCTTTCTTTCCGAAGAAAATATCATCATCCAAATCAGCCCAAGGTCCCGCCTTCACATGAAAACGAAAACTTCTACCATTTTTGAAGCTCACTTAGAGCCGTCCTCCATAACTTGAAGGCTCAGTTACCTTAAAATATTCAGAAGAGAAGAAGAAGCAGAGCAAGAAGATGATGAAAGCTGAAGAAGAAACTCATGATATCGTCATCATCGGCGGCGGCATCTGTGGCCTCGCCACTGCCGTAGCTCTTTACCGGAAGGGCATTAAAAGCTTGGTTTTGGAGAGATCTGATGAACTAAGAACAACTGGTGGTGCCATCTCTATATTTATGAATGGTTGGTATGCGCTTGACCAACTTGGGGTGGGAGATGTGCTCCGTTCAAAGGCTATCCCTATAAAAGAGATAAAGGATTCTTGGCTGTTCAAAGAAGAAACAAAGATAACACCAAGCAGGAAAGAAGGGCTTCGGTGCTTAAAAAGGAGTGATCTTGTCGAAACATTGGCTTCCACTCTCCCAGCTGAATCAATTCGCTTTAGTTGCAGAATTGTAGCAACAGAGATTGATCCAGTCACATCCTTTCCCATTATTCACGCCAGTGATGGGCTCATCATCAAAGCTAAGATTTTAGTTGGCTGTGATGGATCAAACTCAGTTGTGGCAAGAAAATTAGGCCTGAAGGCCCCAAAGATATCCCCCATATGCGAGGCGCGAGGCTTCACAAATTATCCCAATGGCCATAGTTTTGGCGATCAATTTCTGCGTCTATCGGGTCACGATTTCTTACTTGGAAGGGTTCCTGTTAATGAGAAGCTAGTGTTCTGGTTTGTGGATCACAAGTTCAATGAAAGAGATACAGAATCAAGGGAAGATCCCAAATTAATCAGAGATCTAACCTGGCAGCGCCTCGAAGGCTCCCCCGAAGAGGTGATCGACATGATCAAAAACTGCGAAATTGACTCGCTAACACTCACAAGAATCAAATACAGAGCACCATGGCACATTCTATTTGGTAAAGTGCAGAAAGGAACAACCACAGTTGCAGGAGATTCGTTGCACGTAATGGACCCTTCCATTGGACAAGGAGGTTCTGCAGCCATTGAAGACGCAGTGGTGCTCGCCAGATGCTTGGCAGGAGAACTTCAGCCACACCAAACAGCCGACAGCGGTGAACTGAAAAAGAGAGCTGAAGCTGCCATTGACAAATATGTTAGGGAGCGAATGCTGAGGATAATGAGCTTGTCAACACGAGCTTTTCTTATTGGTTCTATGTCTGCAACCTCTTCCTGGATTAAAAGAATGTTTTTTGGTGTTTTGTTAGCTTTCTTCTCGGGGAATAACTCACTTAGCCACGCGCAGTTTGACTGTGGTCATCTCTGAACTTTCGTGGTAGTGATTGGGATGTTCAGAATGAGAAGCAGAAATAATTGTTACAGTATTTGGTTGCTCTATCACGTAAAGTGTAGTGTAGTATTTTGAAATATATGGATTTACACTGCGAGCAATGTTAATAAAGTTTCATCGGGTTGTTGTTTAATATTGTACAAAATAAAACCACACTTGA

>c16388_g1 [organism= *Cymbidium longibracteatum*] chalcone synthase

CATAGACAAGCAGGCCAATTCCAATCTATTTCTTCAATCAAAATACTGAGTTGTAGCAACCAAGGCGTCACTTATCGCTTTTTGCACGATGGCACCAAGAGCCGGCGGCTTCGCCTCCATTTTGGCCATCGGCAGGGCCAACCCCGAAAACGTCATGGAGCAAAGCAGCTTCCCAGATTTTCTTTTCCGTGTCACCAACAATGAGCACTTGGTCGATCTTAAGAAAAAATTCCAACGCATATGTGATAAG

>c4492_g1 [organism= *Cymbidium longibracteatum*] flavonoid 3' hydroxylase

GGCGCGCGGTGGCGCATGCTGAGGCGCCTGTGCGCATCGCATTTATTTTCTGCGAAAGCGATGGAAGATTTTCGGCACGTGCGGGGAGGGGAGGTGGAGAGGCTCGTGCATGGGCTAGCGGAGGAGGAGGGAGTGGCGGTGGATGTTGGCGGGGCGGTGAACACGTGTGCGACCAATGCGCTGACGCGTGTGATGGTGGGGCGGCGTGTGTTCGGGGGAAGGGAGGAGAAAGAAGGGGCGGAGGAGTTTAAGGAGATGGTGGTGGAACTCATGAAGCTCGCCGGAGTTTTTAATATTGGAGATTTTGTGCCCGGATTGGGATGGCTTGATTTACAGGGTGTGGTGAGGAAGATGAAGAAGCTGCATAAAAGATTTGATAAATTATTAGATGGAATAATTGCAGAGCATAGAGAATCAGTAGATAAAGGATCAGTTCATGGCAGAGGCAGCGATATGCTTAGCATACTCCTTCGACTGAAAGAGGAAGCTCATGGTGAAGGAATCCTACTCACAGAAACAGCCATCAAGGCTCTCTTACTGAATCTTTTTACGGCGGGAACTGACACGACTTCGAGCACGGTGGAATGGGCCATGGCCGAGCTAATTCGCCACCCGAATCTCCTGAAACAGGCTCAAACTGAGATCGACTCGGTGGTCGGACACAACCGACTCGTCTCCGAGTCCGACCTCCCCAACCTCCCCTTCCTCCAAGCCACCGTCAAGGAGACCTTCCGCCTCCATCCCTCAACCCCGCTCTCCCTCCCGCGCGTCGCTTCGAGCGACTGCGAGATCGACGGCCACCTGATTCCTCGCGGCGCCACTCTTCTCGTCAACGTCTGGTCCATTGGGCGGGACCCGTCGATGTGGCCCGACGAGCCGCTCGCGTTCCGGCCCGGACGGTTTCTCGCCGGCGGCCGGCACGAGGGAGTCGACGTGAAAGGGAATGATTTTGAGCTCATACCGTTCGGCGCGGGGCGGAGAATCTGCGTCGGGCTGAGTTTAGGTTTGAGGATGGTTCAATTCATGACGGCGACGCTCATTCATGCCTTCGATTGGGAGTTGGCCGGCGGAGAAACGGCTGAAAAGCTCGATATGGAGGAGGCTTATGGGCTTACTCTTCGCAGGGCGGTGCCGCTCATGGCGAAGCCGACGACTAGGCTAGCCCTAAAGGCATATCCTAAGGATGTCTAATACATGTAGCTTGTTATTAAACAATATTTATGAG

>c52282_g1 [organism= *Cymbidium longibracteatum*] flavanone 3-hydroxylase

AAAACTGCTATATTTATAATAATAAAATCCTTTTCTTCCTCCAACCATTTCCTCAATCGACATGGAGGTGGAAATACAGAGAGTCCAATCCATCGCCTCCCTCAGCCTCGACACGATTCCGCCGGAATTCATCCGGTCGGAGGAAGAGCAGCCGGGCCTCACGACCTTTCAAGGACCGGTTCCGGAGATCCCGGTGGTGGATATCGGCGGCGGCAATGAGGAGAAGGTGACGGAGGCGGTGGTGGAGGCGGCGAGGGAGTGGGGGATATTCCAGGTGGTGAACCATGGGGTTCCGGCGGAGGCGGTGAGGAAGCTGCAGAGAGTGGGGAAAGAGTTTTTCGAGTTGCCTCAGGAGGAGAAGGATAAGTATGCGATGAAGGAAGGGAAGCTTGAGGGATACGGGACTAAGCTTCAGAAGGAGGTTGCCGGGAAGAAGGCTTGGGTTGATTTTCTGTTTCATAATGTGTGGCCGCCCGCAAGTATTGACCATCGGGTCTGGCCCGAAAACCCGCCCGATTACAGGAAAGTAAATGAGGAATATGCTCAATGCCTTGTCACTGTGGTAGAAAATCTATTGAAGTGGCTTTCTAGGGGACTAGGGCTTGAAGGTCATTTACTGAAGATGGCATTGGGTGGTGATGAGATGGAATATTTACTCAAAATCAATTATTACCCTCCATGCCCCAGACCTGATCTGGCTTTGGGTGTGGTGGCCCACACTGATCTATCTGCAATTACCATTTTGGTCCCCAATGAGGTCCCTGGCTTACAAGTTTTCAGAAATCATCACTGGCTTGATACTAACTATATTCCTAATGCTCTCATTATTCACATTGGTGATCAAATTGAGATCTTAAGCAATGGGATATACAAAAGTGTGCTGCACAGAACGACGGTGAACAAGGAGAAGACGAGAATGTCATGGCCCGTTTTTGTTTCGCCTCCGCCGGAGAAGATCATCGGCCCGCTGCCGGAGCTGCTCGGCGGCGAGAATCCGGCCAAGTTTAAGTCCAAAAAATTCAAGGAATATCAGTACCGCAAAATCAACAAGCTGCCGCAGTGATATCTGAGAATAATCAGGCTTAAGTAATTATAAGTACATTTTAAGTATAATTAATTTGAGTTATACCTATTGAGATCGAGAGCAATGTTATCTCTCTAAATTAACGACTTTAGTACAATCTTTATCTTATGTGTTGATGGTTGTT

>c78740_g1 [organism= *Cymbidium longibracteatum*] Caffeoyl-CoA O-methyltransferase

AAATTTAGAAAACCTACTTACCTTCGGCAATATAACTGTGTCGCCTTTCTCAACCTCTTTTACCGAAGCCAGGAAGAACTTTAGGTGAAGCAGTTTCTTTTCAATGGGTTCTGATCTCTCTGGCATCAACAAGTGCTTGTTGCAAAGCGATGCTCTTCAACAGTACATTTTGGAGACAAATGTTTACCCAAGAGAGCATAAACTGCTTAAGGAATTGAGAGAAGAAACTGAGGCTAAGATTCCCAGGAGGGCTTTGATGAGTGTGCCCCCTGATGAAGGGCAACTCCTTTCTATATTGCTCAAGGTGATGAACGCCAAAAAAACCATAGAACTTGGAGTTTTCACTGGTTACTCTCTCCTGACTACAGCTTTAGCCTTACCGAAAGATGGAAAGATTACAGCGATCGATATCGATAGATCATACTATGAGATAGGCTTGCCTTTTATTCAGAAAGCAGGAGTTGAGAACAAGATTGAGTTCATTGAATCAGAAGCTCTTCCTGTCCTAGACAAATTGGTTGAGAAGGTTAAGGAAGATGATTTATATGATTTTGCATTTGTGGATGCTGACAAGACTAACTACCACAAGTATCATGAGAGGCTGCTGAAACTAGTCAAAGTTAACGGTCTGATTCTGTATGATAACACGCTCTGGTTCGGAACCGTCACCGGCCCGTCAAGCCCAAACTATCCTGATTGGATCAACTATAACCTGGAGAGCATTAGACAGCTAAACAAGCAGCTGGCCGCAGATCCGCGGGTTGAGCTCTCACAGGTCTGCATTGGTGATGGAGTCACCATTTGCAGGCGCATCTCCTGAGCTAAGTTATAGAAAACTGAAACCTTGAAGGAGGAGACCATTGTTATGATTTGGTTGTTTCTTGATGCCTGAGGCATATACATAAGTATTGGTGTATCATCTGTTCCTAGATGCACTTATGGCTTTGTTAATTTGTTATGATCTTTTTTCTTAGTTCAAAACTGTTTTCTTACTTCAATAACAATTCACAACATTTGTGCTGTTAAATGTTTGTGTTTCTCTACATGTTATCTTCTGCTGAATGAGTTGTTATGTCAAAAGTAGGGGTGTAAACGAGCCGAGCTT

>c42659_g1 [organism= *Cymbidium longibracteatum*] R2R3-MYB transcription factor

AACTGATAACGAGATCAAGAACTATTGGAACACACATCTAAAGAAGCAGTTAGCAAAGATGGGGATCGACCCAGTGACACATAAACCCAAGAGTGATGCCCTCGCCTCTGCGGACGGCCACACAAGGAGCACTGCTAATCTCAACCACATGGCCCAGTGGGAGAGCGCCCGCCTTGAGGCTGAGGCTCGCCTCGTGCGTGAATCAAAGCTCCGCAGCAGTGCTCCTCCCTCCCCCTTCCCTCCGCAGTATCTACCTCAACCACCACCTCCTGTCTCCATGCCGACAGCAGCATCTCCCTCTCTTGACGTACTGGGAGCTTGGCAGGGTGAGTGGCCCAAGCCTGTAGTCAATAGTCAAGCAGGCAGCCACAACATTGACCTCGAGTCGCCAACCTCCACTCTGAGCTTCTCAGAAAACATGTTGCCATCAAGAATCCCAGGCATGGGGACTGCAAATGACAGCACAAACTGGAAATGCCTCAAGAAGCCAGGCTTCTCACTGGACACAGCAGAAGCTTTTGTTAATGCCGAAGCAACTTCTTGGCTTACCGGATCCTGCAGTGGAGGATTTGCAGCCGGCTTCACTGGAATGCTAATGGGTAACACAAATAAGCAGAACTCAACAGAAGGTTGTGATGATTCTGACATTGCAGGTGGCAGCTGCGTGGATGTGGAAGAAGGTGAAGATGAAGCGGAGGAGAACAAGAACTACTGGAACAGCATATTCAACTTGGTCAATTCGTCATCTCCCTCAAATTCGCCGCCAGCTGTGTTCTAGTAATCTGAACATTAGCACCTAAGTTCTCAGGTAAGCTGTATCTAGCCCATTATCATATGATCTCGATTCTGCTTGAAGTCAACCTTCTTTTTCAATCCCTGTAGTTTCCGTTTTTTTCAAAAAAAATTGTTCAGTGTGGCAGCTAGCTGAGCCGACCCAATTCATCCTGAAAAGAAGCGATTTAACTGAAGCTCCTGAGTTGACTCGGTTATCATCCCTCTGCTTTTATTAGGGTAAATCTAGGTGCAATGCCAGCAAAATTGAATAATCAAGGTGGAAAGGGCAAAAAAAAAAAGGAATTAAACAAACTATTTTTAATTCTTACGTGCTCTACTGTGTAGAAT

>c51607_g1 [organism= *Cymbidium longibracteatum*] transcription factor bHLH76-like

CTCCATTCTTCTTCTTCTCTTAACTTATGGCGCTTGCTAAGGAGAGAGCAATATATAGTTCACAGTCTAGTTTTATTCAAGGGATTGATCCTTCTTCGCTCTCTCACTTGTGCTTCAAAGATCATCAGGGTTTTCTTCACGATGAAATATACTCTAATTCGAACGCTTTTGTGTTTGAGGCGTCGAGTTTCCCTCTGCAAGAAGCTCATCCTCTCATCAACTTTAAAGCGAGCGTTGATCATCAGTTGAGGACGTATTCTGCTTCGACGGTTCTGAGCTTCGAGCGGAGTCTCGATTATGGAGAGAATTCTGTGAGCAGTTGGATCGATGCAATGGACGATGAGAACGATGTGATTAGTCATCTTAGCTCTAGGAGTTGTGAGATAAATCGTTTCAAGAATGAAGAAGAGAGAGTTGGGAAGGAGAGAGGATTGAGCAAGAGGGCATTAATGGAATGTGATATGGAAGCTTTAAAAAAGCATTGTGGTAACACTAAGAGGCAAGTCAAGTGTAAGATAAGTTCATCAAAGGATACACAAAGCATTGCAGCAAAGAATCGCAGAGAGAGGATAAGTGAAAGGCTTAAAATTCTACAAGCTCTTATCCCAAATGGCACAAAGGTTGATTTGGTTACAATGCTGGAGAAAGCAATAAGCTATGTGAAATTCCTCCAACTTCAAGTAAAGGTGTTAGCCACTGATGAGCTCTGGCCAGCTCAAGAAGGAAAAGCACCTGAGATTTCTCAAGTGAAGGAAGCCATTGATGCAATCTTATCTTCTCACAGAGGCAAATAAGCTCTTCAAAAACTGAAAATTGAGGGACTAAAATGAAAATAATTTTATATAAAGTGTTGTAGGAGAAGCCAAGACCTGCAGAGGAAGTGGACGGATTTTATTTTTTATTTTTTATTT

>c82816_g1 [organism= *Cymbidium longibracteatum*] transcription factor bHLH78-like

CTCATTCTCACTCTTTTTCCTCTGCTCTACTTAATCTCAGAAAACTGAGCTATTATCAGAGGCGGTTTCTAGCTGCAGAGGAATCCATTCCTCTCTCTTTCCTCCTCCTCCTGCAAAACTGCTACTACTTCTACAAAAGCCATATTAGTCAACTTTACCATTCCTTTCTTCTCTGTCATCCACCTCCTTCCCTTTCTTCAAAGCCTACGCTTTCTTCCCTTTTCGCCCCAATTCTCATCCAAAGCTACCTGCTTTAGTTTTTCTTGTCGAGCCCTTTTTATTAAATTCGAATCCGGTTCTTTTACTTTTACACACTTCTTTAAACCTTCTTATCGAGAGCTCCAATTCTGTAAAGGCGAAGACTTTCCCCGACCTCCGTTGGAGACATTCCATGGACCGACTCACCCCCATCACTCCCCTGCCGATGGCGGCCTCGACAGCCATCGCCGGCAATATTTCCAGTTACATAAATAACTCTCTGAGCCTCTTGCCTACAAACTGTTTGATACCATTTCCCACTGATTCCGGATTCACGGAGAGGGCTGCGAGATACTCCTCGTTCAGTTCTGACAACTACAAAGAAGCTCAAGGTAGCATCTTGGACACCGGTAAGCTCTCGAGGGTCTCGAGCAGCCAATCCCTGAAGTCAAAGGAAAATTCGTCTGTGACGGATCTTGGTTCGAGAGCCGGAGAGTGTAATGCCAGGAAGAGGAAGGCAGCGCCGAAGAACAATGCTAAGGTGGCTGAGGAGAGTAATATGAATACAAAGAAATGCGGGTCCGCAGAGACTAGTGATGAAAAGTTTGACTCTCAGCTTAACGAAGTTCGGAATGGTGAGGAAAAGAAGGGGAAGGATAATGGCTCAGAACTCCCAGAGCCTCCCAAAGGCTACATCCACGTCAGGGCAAGAAGAGGCCAGGCCACTGATAGCCATAGCCTTGCTGAAAGGGTTAGAAGAGAGAAGATAAGCCAGAGAATGAAAGCGCTTCAAGATCTTGTGCCAGGTTGCAACAAGATAACTGGTAAAGCCGTCATGCTTGATGAGATAATAAACTATGTGCAGTCATTACAGCGGCAAGTTGAGTTCCTTTCAATGAAGCTGGCCACTCTGAATCCCCGTTTAGACTGCAATTTGGCTAACCTCGTCTCAAAGGAGTCTCTCGTGAACCAAACAAATACTTATTTACCGAGCTCGGTTTACCCCTTTGACAATTCAGCTGCTACATTTCCATCTACACACCAGTCCAACGAGGGAAACCCTCTACATTTTAATGTGATCAAAGGCATGGAAACTCACATCTCTTTGAACCAGGTCAATTATACTCTCTATCAAACTCCATACCTGCAGCCAACTTCCATCGATGTTTTTGGAGCTCCTGCTTCTCTGCTCGGAAATCCTTGGGATGATGATCTTCATAGCATTGTTCAAATGAACCTTGTGCAGAACCAGGAGATTGGATTCACCTCTCAGAACGTTCATGGGCGATTAGCAGCAAACAAGATGAAGGCTGAGCTCTGATTGCGTTTGGTTTCTTTCATAATTGAAGATCTCTTTATATGTATATAGTAATTTCAACTTAAGTGCGCAGATAGAATGAGTAAGCTGACAGTTTTTAGTCATCCCTGAAACATTCATCTGCTCTTTTTTTTTCTCTTCATTAAGTTAATGATTATTTCTAGGAGTCTTGTAAGATCTTCTAATTCTTATGGCAATAGCATCCTGTGATGCAATGGTGTCTGAAAACCACAATTAATATGTAATATGGTTTGTGCGAAAGAAATAAGAGCTATTGAGATTTTTTTTTGTTAATTACCG

>c80144_g1 [organism= *Cymbidium longibracteatum*] NAC transcription factor 25-like

TATCTTAAGAGCAGAAGTCAAACAGGTTGTTAATGTTATTAAGATGAAATGTCTCAAAGTTGATTCAATTTGTGTTTTCCCTGAAATTGAGACTTAGATATAAAATTGCACATGAGCTTAAGATATTAGACTTAAAAAAGGAGGCTAAGGGAGAAGAATTTTGCTCATAGCTTCTTCTCCATTATTAGATTCTTCGTCGGAGTGATCATCGGTAACGCAGCTCGTGGAAGAAGATGAACAAGTTGCAGGAATTTGAAGGTTTTCTTCTTCATGCCTCGTGTTCCTTGTCCTCAACTTCATGTTTCTTCTCTTCACAAAGATTTGGCAAACTACCCACTCTTTGCTTGAAGCAAGCAAACTCTGCAACCATGAAAGAGCACAATATTCATGCATGAGTCTGAAGAAAAATCACCATACTATCAGTGATCTAAAAGGGTTATCTTTGTCAAATATTTGATGAACTGCATAATCTTCATAAAAAAAATAGAATATTTTTCTAAGCTATAGTAATTCGCACTTACTTGGGTTGAGCTCTTTAGGAGTTCATTGTTGGATAGGCTAAACTCATGCTTGATCCAATCTGTTTTTGATCCATGAGGTGGCTTCCCTCTGTAAAAGACCATGACTTGTTTCAAACCAACTAATTCTTTGCCTTGAGAAGAAGCCAGGACAGGTTTCTCCTTCCCCAAGGGTTTCCAATAGCCCAATCTTGTTTCTTGGTACGATACTCTCCCTTTCTTCTTCTGACACTTACTCTCCCTAAGATAGAAGAAGTATTTCTCTCCACCAAAGCCACCTAAGTTTATTACAACATAACGACCCAAATGTACTTCAAGAAGGGAATATGTTTTATACAAGAAGTTCAAACAATATGATCAAAATTGAGGCTTTTATACCAATATGCTCACAAAATAACAAAATCTTTCTCGAAGAAACTTACAGGGTAAATCCCATGGGTTCAGTTTTCCGAGCTCGATCTCGGGAATGATGGCTGCAGGCAAGGGAAAGGAGAAGGCCTTTCTTCTCAGGTACTGAACAACAAGCTCTTCATCAGTAGGGTGGAATCTGAAACCAGGGGGCAGCCTCACCAGACCATGGTTCATAAACTCTTTTCTCTCCATTTCTATCTCCACGAAGTAAAAGACTCCTCTACTTTGTTTCTCCTCAGAGGACACTCCTCGAGGCCCAAATTGGCTACCTCAGTAGAATTCATCATCCATTTTTAAAGGAGCTAAGCGAAGGAAAGACGGTGAACGAAGAAGAGAAGAGGCAAGTAGATGTGAGTTGCATGGGAGAGGGGGAAAGGGGAGGTTCTTTATAAGAAAAGGGCAAGGAGAAAGGTAGAGCTGTGATAGAACGCAATGGCGTGAGCTACACTCAAGTAAAGAAGCCGCTTTCTTTCAATACCCTCTTCTTTTGGCAACTACGGGAGGGTTGTGTGGGATAATTTACGCCAATGCGTGGGAGTTTTTTGCATGAAACTTTTGAAATGTCGCACATGTCATGCTCTTCAAGCTTAGCAGGGTTAGGAAGGAACTTATTTTTTCAGATA

>c101297_g1 [organism= *Cymbidium longibracteatum*] Putative uncharacterized protein

AGGGCCAGGGGCTGCACCGCGCAAAGAAGGTGCTTTCCCTCCGCCGCCCCTTCGCCCCGCCAAGGAACAACTTTACCGCCGCACTGGTCAAAACCCCACGTTTTATATATCAATCCATTCTCTCGGTCCGTAAATTGGACTCCTCAGATTACTCCTTAATGGCCACCGCAGCCGCCGCTGCCTCGCCGGCGACATTCTATGGTGTTAGCCTCCGTTCGTCCACGAAATCTATAACCCACTCTTTCATTCGATCTCACCGCTTTTTCGTCGCTCTAAGTTCCTCCTCCTTTCCGTCTCCATTCACAGAGAGCACTTCTTCTGAGAGATGCCGGAGGGACAGCTGGGCGTACGGCTCCGCTGACCTTGCGGTTGGCTTTCCTTCTTCCCCGCTTTGCGACAAGGAGAATGATATCGCTGTCCAGCTCCCCGAGCTCAAGCGACTACTCGAGACCCTCAAAGCCGCCAAAAAGTCAAGCGGTGGCGGCGGCAGAGGAGGAGGAAGGCGACCAGGGAGTGTGACGCTGGTCGGAACTGGGCCCGGAGACCCGGAGCTACTGACCCTCAAGGCTGTTTGGGCGATTGAGCGGGCCGATCTCATTCTCTACGACCGCCTAGTCTCCAACGACGTGCTGAGTCTAGTTAGAGGGAATGCTAGGCTTCTCTACGTCGGCAAGACTGCTGGGTATCACAGCCGGACGCAGGAGGAGATTCATGAATTGCTGCTTAATTTTGCGGAGGCTGGTGCTAATGTCGTAAGGCTGAAAGGAGGGGATCCTCTGGTGTTTGGTAGAGGTGGAGAGGAGATGGAATTTTTGCAACAACAAGGAATAGAAGTAAAAGTTATTCCAGGGATAACTTCTGCTTCTGGAATAGCAGCAGAGCTTGGGATTCCATTGACACATAGAGGCGTTGCAAATAGCGTTAGATTCTTGACCGGTCACTCGAGGAATGGTGGCACTGATCCCTTATATGTGGCTGAACATGCTGCTGATCCTGAGTCAACTTTGGTCATCTACATGGGTTTGTCCACTCTTCCTGGTCTGGCCTCTAAATTAATGAAGCATGGCTTGTCACCTAAGACTCCTGCTGTAGCTGTAGAGCGAGGGACGACTCCTCAGCAACGAACAGTGTTTGCTGAGTTGGAAAACCTT

>c59163_g1 [organism= *Cymbidium longibracteatum*] Glutamyl-tRNA reductase 2-like isoform X2

GGCGGGGTGAGACCCAAATTCTCAAATATCATCTTCACCACGCGCCCAACGATGCGCTATATTTTTATAATTCCCGGCGTGTGGTTCCCACCTCCACCCACCCACCCACCGGCCACCGCACGTTTTCGGTCGCCTGTACGAGTATACGACCTAGCTAATGTCAGCTTCCTGCCTGCTCCATTACTAAGAAGTTTGATGTCTTCAGCTATATAAAGATCTCTCTTTTATCTCCGCCAAAAGTTCCCCATAGGGGCGGAATAGAGAGGAAAGACGACCGGCGGCAAGCGGCAGACTGGAAAAAGAGAAAGATCAAGTCTCTTTTTCTCTTTCTCTCTTCCTATCCCTCATATATCGCAGAAACCTTGATCTCCTCGGAGCCGGCGGCTGCAGTTTCTGATCTGTCCCATGGCTGTTTCGTCGACCACGGCCTTCGCGGCCTCTCTCGCGATCACGAAGCCTGATTACTTAATGTTTCTCAGGAGGCCGGCTGGAGACTTCGGAATACGAAGCATTTCGTTGCCGACAAGGAGCCGGAGATTAAGCAGATTTGGGGCTAGATGCGAGGCGGTGACGGATATTGGTGTGAAGAACGCTTCCGAATCTAGGGCTTCAAGTATCTCCGCGCTCGAGCAGTTCAAGGCCCTCGGCACTGATCGATATATGAAGGAAAGGAGTAGCATTGCTGTTATAGGACTTAGCGTTCATACTGCGCCCGTGGAGATGCGAGAAAAGCTTGCTGTTCCTGAGGCTCAGTGGTCCCGTGCCATTGGAGAGCTATGCAACCTTAACCATATTGAGGAAGCTGCTGTTCTCAGCACCTGCAACCGAATGGAGATCTATGTGGTCGCTCTATCTTGGAACCGCGGAATCAGAGAAGTGATGGAGTGGATGTCAAAGACAAGTGGGATTCCTGTTTCAGAGCTCAGAGATCATCTCTTTATGCTGCGTGATAGTGATGCTACAAAACATCTGTTTGAAGTTGCAGCTGGCCTTGATTCTCTTGTCTTAGGCGAGGGCCAAATCCTCGCTCAAGTAAAGCAAGTTGTTAAGATTGCCCAGTCAAGTGGAGGACTGGGGAAGAACATTGACAGGCTTTTCAAGGATGCGATCTCCACTGGGAAGAAAGTTCGCACCGAGACTAACATTTCTTCCGGCGCAGTCTCTGTGAGCTCGGCCGCCGTGGAACTGGCTTTAATGAAGCTTCCAGCTTCCCATGCCTTATCTGCCAGAATGCTTCTGATTGGTGCTGGCAAGATGGGGAAGCTTGTGATCAAACACCTAGCTGCTAAAGGTTGCAGAAGAATCATAGTCGTTAACCCTTCGGCGGCGAAGGTCGATGCTATTCGCGAGGAACTGGAAGATATTGAGATAATCTACAGGCCTCTTGCTGAAATGTTCTCCTCTGCAGCGGAAGCCGATGTTGTTTTCACCAGCACTGCGTCCGAGACGCCATTATTCTTGAAAGAACACGTCGAAAGCCTTCCTCCTGTGAGCGAAAGCGTCGGCGGATTCAGACTCTTTGTTGATATATCTGTGCCCAGGAACGTAGGTTCATGCGTTTCTGATGTCAAAACCGCACGGCTATACAATGTTGATGACCTGAGAGAGGTGGTGGAAGCCAACAAGGAAGACAGATTGAGGAAGGCGATGGAAGCCCAAGCCATAATCACACAAGAGTTGAGGAGTTTCGAGGCGTGGAGGGACTCGCTGGAGACCGTACCGACGATCAAGAAGCTGAGGTCCTACGCCGAGAGAATCAAAACCGCGGAACTCGAGAAATGCATGCAGAAGATGGGCGAAGATGTTTTGACGAAGAAGGTGAGGAGAGCTGTGGAAGATCTCGCCAGCGGCATCGTGAACAAGCTTCTGCACGGTCCGCTGCAGCACCTGCGATGCGATGGCACCGATAGCAGAACTCTCGATGAGACGCTGGAGAACATGCATGCGCTGAACAGGATGTTCAGTCTTGATACAGAAAAAGCCATCTTGGAGCAAAAAATCAAAGCGAAGGTGGAGAAAGTTCAGAGCTGAACAAACTGATGAAAGAACT

>c19225_g1 [organism= *Cymbidium longibracteatum*] Chlorophyll a-b binding protein 13

GTTGGAGATTCTCCAAATCCTTCTCCGCATTCTCATTGGTCCAGAAACAAACACAGCAATCTCAGTGCATACCTCTCACTCCACTCACCAAGTCCTTAAACTACCACTCTCACCGCCACCTGATAGAAACACAATCACAATTTCTCCACAACTTTGAAGGAAAAAGAATCACAATTCAAAGAGGGTAATTCAGCCACAGAGTCTGATGGCAGCCACCATCATCAAGCCAACTCCATTCCTTGGTCAGGCTCGATCGTCTTCATCCGCCATCAACCCTCTCAGGGATGGCCTGCAAATGGGAAATGGAAAGTTCAGAATGGGGAATGAGCTGTGGTATGGGCCTGACAGAGTGAAATACTTGGGACCTTTTTCTGCCCAGACACCATCATACCTTAATGGAGAATTCCCCGGGGATTATGGTTGGGACACCGCAGGGCTGTCGGCTGACCCTGAGGCATTTGCCAAAAACCGTGCTCTTGAGGTAATCCATGGGCGATGGGCAATGCTTGGAGCTCTCGGATGCATAACTCCTGAAGTTCTGGAGAAGTGGTTGAGAGTTGATTTCAAGGAGCCAGTTTGGTTCAAGGCTGGCTCCCAAATCTTCAGCGAAGGAGGCCTGGATTACCTCGGCAACCCCAACCTCGTCCATGCACAGAGCATCCTTGCTGTTCTCGGCTTTCAGGTAGTCCTCATGGGACTTGTTGAGGGCTACCGCATCAATGGCTTGGATGGAGTTGGAGAGGGCAACGATCTCTACCCCGGTGGCACCTACTTTGACCCCCTCGGCCTTGCTGACGATCCTCCAACATTTGCAGAGCTGAAGGTGAAGGAGATTAAGAATGGCCGGCTTGCAATGTTCTCCATGTTCGGTTTCTTCGTTCAGGCCATTGTTACAGGGAAAGGACCGTTGGAGAACCTCTTAGACCATCTGGATAACCCTGTGGCTAACAATGCATGGGCTTATGCCACCAAATTTGTGCCTGGCTCTTAATTAACAGAAGGTTTTCTGGAGCTCTTGTGGATTGTTATGTCCAAGCTTTGGAAATTGAAATTGTACTGTGAATTATGCTTATGATGAATCAATGATGATTTTCATTTCTTTGCTTGGATGTGGTTATTTTTTTGTTTTAATTTCTTTCACTTAAGAAATTTGAATTTCAAAGACTTTAGATGAA

>c40965_g1 [organism= *Cymbidium longibracteatum*] Chlorophyll a/b-binding protein

CCTCTCAGGCCAAACTATCAAATTTACATGGACGATATAAATAAAACAGTTCAGAAGAACCATTAGTTAGCAGGCCCGAGTCAATTAAGTCTACATTCAACTCCTCACTTTCCGGGCACAAAGTTGGTGGCATATGCCCAAGCGTTGTTGTTCACCGGGTCAGCAAGGTGGTCGGCCAGGTTCTCCAGAGGTCCCTTGCCGGTAACGATGGCTTGGACGAAGAAACCGAACATAGAGAACATGGCGAGTCTACCGTTCTTGAGCTCCTTCACCTTGAGCTCAGCAAAGGCCTCTGGGTCATCGGCCAAGCCCAGAGGGTCGAAGCTGTTGCCTGGGTAGAGTGGGTCAGTGATCTCGCCTAAAGGTCCTCCTCCAATACGATAGCCCTCAACAGCTCCCATGAGAACAACTTGAACAGCCCAGATAGCCAGAATGCTCTGAGCGTGGATCAAGTTCGGGTTGCCTAAATAATCCAACCCACCCTCGCTGAAGATCTGAGACCCGGCCTTGAACCACACTGCCTCACCGAACTTGACGCCATTGCGAGCCAAAAGCTCTGGGAACACACAACCGAGTGCACCCAACATAGCCCACCGTGAGTGGATTACCTCCAGCTCCCGATTCTTAGCGAAGGTCTCTGGGTCGGCTGACAAGCCGGCAGTGTCCCACCCGTAATCGCCTGGGAACTCGCCGGTAAGATAGGAAGGTGGCTCGCCGGAGAACGGGCCGAGATACTTTACACGATCCGGCCCATACCATGGGCTGCTAGTTGAGGGACGGAGCTTACCTGCTGTAGTGCGGGGCCTGCCCGATGTAGTCTTACCCTGCATGGTGATGCGGCCCTCGCCAAAGATGACAGCGGTAGAGGGGGAGACTTTGACGGCTTTACCGGTGAGGGAAGGGGATGAGAGCGCCATTGTGGCTGCGGTGGCTGCCATTGTTGGTGTTGGAAGTAGTAG

>c101481_g1 [organism= *Cymbidium longibracteatum*] RuBisCO large subunit-binding protein subunit beta

AATTACAAATGCAAGGGATTTGATCAATGTCCTGGAGGATGCAATTAGAGGGGGATATCCTATCATCATAGTTGCAGAAGACATTGAACAGGAAGCTCTGGCAACTCTTGTTGTAAATAAGCTAAGAGGTGCTTTGAAGATTGCTGCTCTAAAAGCTCCTGGATTTGGAGAGCGCAAAAGTCAATACCTTGATGATATTGCAATCCTTACTGGAGCAACTGTCATCAGAGATGAGGTTGGCCTTACACTGGATAAAGCTGAAAAAGAGGTGTTGGGTACTGCTTCCAAAGTTGTGTTAACCAAAGATTCAACCACCTTAGTAGGTGATGGAAGTACACAGGAAGCAGTAAATAAGCGAGTTGCACAAATTAGAAACTTGATTGAGGTAGCTGAGCAGGATTATGAGAAAGAGAAACTGAATGAGAGGATTGCAAAGCTCTCTGGTGGTGTTGCCGTCATTCAGGTTGGAGCACAAACAGAGACAGAGCTCAAAGAGAAGAAGCTGAGAGTGGAAGATGCACTGAATGCGACAAAGGCTGCAGTTGAGGAAGGTATTGTTGTTGGTGGCGGCTGTACCCTACTGAGGCTGGCTTCAAAGGTTGATGCCATTAAGGAAACTCTTGAAAATGAAGAGCAGAAGATTGGAGCAGATATTGTCAGGAGAGCTCTAAGCTATCCTCTTAAATTGATTGCAAAAAATGCAGGTGTGAATGGAAGCGTTGTGATTGAGAAGGTGCTTTCCAGCGACAATCCCAAGTATGGCTACAATGCAGCTACAGGAAAATATGAGGATTTAATGGCTGCAGGA

>c39290_g1 [organism= *Cymbidium longibracteatum*] Chlorophyll a-b binding protein CP26

GACATCGTCTCCGACCACGCTCACTCCACATCCCATCTTCTGGCGGAAATTCTTCCGATCAATGGCTTCCCTTGCCGCTGCAGCTCCAGCCGCCCTCGGCCTTTCCGAGATGTTTGGCGTCTCGCTTGGCTCAATCGCCACCGTTCGTTCTGCTGCTCCGCCTTCTTCCTCGGCTGTCGGCTCTAAGATTGTCGCTCTCTTCTCTAAGAAAGCTCCGGCCAAGTCAAAGGCCGCCGCCGCCGCCGACTCCCCCATCAGTGAAGAACTGGCCAAGTGGTACGGTCCTGATAGGAGGATCTTCTTGCCGGAGGGGCTGCTTGATCGATCGGACGTACCGGAATACCTCACCGGAGAAGTGCCTGGAGACTATGGCTATGATCCCTTTGGTTTGAGCAAGAAACCAGCTGACTTTGCCAAATACCAAGCCTTTGAACTTATCCACGCAAGATGGGCAATGCTTGGAGCTGCTGGCTTCATCATCCCAGAGGCCTTCAACAAATTCGGAGCAAATTGTGGCCCAGAAGCCGTATGGTTCAAGACTGGAGCTCTCCTGCTTGATGGCAACACATTGAACTACTTTGGCAAGAACATTCCCATCAACCTCGTCCTCGCCGTGGCAGCCGAAGTCATCCTCGTCGGCGGAGCAGAGTACTACAGAATCACCAATGGCCTGAATCTGGAAGACAAACTTCACCCAGGTGGACCGTTTGATCCTCTCGGCTTAGCCAACGATCCAGACCAGGCGGCGCTGCTCAAAGTGAAGGAGATCAAGAACGGGCGGCTCGCCATGTTTGCTATGCTCGGATTCTTCCTTCAGGCTTATGTCACCGGCGAAGGGCCGGTCGAGAACCTCGCAAAGCATCTCAGTGATCCTTTCGGCAATAACTTGCTCACTGTTATTTCTGGCACTGCTGAGAGAGCTCCTACTCTGTAGTTT

>c100760_g1 [organism= *Cymbidium longibracteatum*] Pleiotropic drug resistance protein 12-like

CTCCATGATGAGTTTTCAATGGACGGCCGCAAAGTTCTGTTGGTTTTATTTCATATCTTTCTTCTCCTTCCTCTACTTCACCTACTACGGGATGATGACCGTGTCGATCTCGCCGAATCATCAAGTTGCTGCCATCTTCGCCGCGACATTTTATTCGCTCTTCAACCTTTTCTCCGGCTTTTTCATTCCAAAACCCAAAATTCCCAAATGGTGGATCTGGTATTACTGGCTTTGCCCAGTGGCGTGGACGGTTTACGGGCTAATAACGACCCAATATGGTGATTTGGATCACCAAATCCGTGTTCCTGGGCTGGGTCAACAATCAATTAAAACTTATGTTAAAGATCACTATGGTTATCACACTGACTTCATGCCTGTTGTGGCTGTTGTGTTGGTTGGTTTTTGTGTGTTCTTTGCCTTCACCTTTGCCTTCTGCATCAGGACATTAAACTTCCAGCAGAGGTAAAAGCCAGAGATGTAGAGATCATTCTATATATTTTATATGTTTCCAGTTGGAATTTTGTATACTCTCAAGTATTTTTTTTTCTTTTTTTAATTTCCTGTAAATATTCATATAGAACGAAG

>c110275_g1 [organism= *Cymbidium longibracteatum*] Photosystem II 10 kDa phosphoprotein

TTGTAAACCACGATCGAATCTATGGAAGCATTGGTTTATATATTCCTCTTAGTTTCGACTTTAGGGATAATTTTTTTCGCTATCTTTTTTCGAGAACCACCTAAAGTTCCAACTAAAAAAATGAAATGATTTTTCATTATTTCCATTGAAGTAATGAGCCCCCATATTAATATTGGAGCTCATTACTTCAACTAGTCCCCATGTTCTTCGAAGGGATCTCTTAATTTTTGAGAGGGTTGCCCAAAAGCGGTATATAAGGCATACCCAGTAAAGCTTACAAGTAAACCGGATATGGAGATGGCGACTAGGGTTGCTGTTTCCATTTTTCGATAATTTCAAGATCACAAAGGATCACGATAATGTTGTTTATTTACAACTACAACGGAATGGTATACAAAGTCAACAGATTTAAACCCATGATGAAAGAGGATTTATGGCTACAAAAACCGTTGAGAGTAGTTCTAGATCTGGGCCAAGATCAACTGGCGTAGGGAGTTTATTGAAACCATTGAATTCGGAATATGGAAAAGTAGCTCCAGGGTGGGGGACTACACCACTTATGGGAGTTGCAATGGCTCTATTTGCAATATTTCTATCTATTATTTTAGAAATTTATAATTCATCTGTTTTACTGGATGGAATTTCAATTAATTAGTTCATAAAAACTAGTAAGTCCTAGCAAAAAGATTATTTTACTTATACTTACTTAATGCTTAAGATACTGAATACTTCAACTTAAGATTACCTAAGACTTGGATTTATAGACCATTCTGGTAGTTCGATCGTGAAATTTATTTGTTTCGATATTTCATTTCCGGAATATGAGCGTGTGACTTGTTATAATTGATCCTATTGATAATACAGAGAATGGACCTGTCATCTCTATCAAGATGATTCTACCTCGTCAGATATTTATTCTAGTCTCTGGAGCACGGACTATATAGAATAGATCAAGAAAAGAAATAGTTGAACTATGATTCATACCTATTATTCAGACCTCGCAACCGGATTAAAAAAAAAATGGAAATAGGGAAATAGGTCTTTTCTAAATCAAAC

>c111436_g1 [organism= *Cymbidium longibracteatum*] Alcohol dehydrogenase 1

GGCACGTGGTTTCGTCTCCGTAGAACCCGTCGCTTGAGAAAACAACAGCTAAGAGAAAACGACAGGATTCAGATCTTCACTTTCAAATTATAAATTTCTCACAGCGCCTAATGATCTCTTCATTCTACAACAGTTCTTGCAATTTTTGTTTTTCTATTTTGAAGAATCGTTTGATTGTGTTTTGAGCTGCAATGGCGAGCACTGCGAGCAATCCGATCATCTGCAATGCGGCGATTGCATGGGAGGCGGGTAAGCCGCTGGTGATCGAGAAAGTGGAAGTGGCGCCGCCCAAAGCGATGGAGGTTAGGGTTAAGATCAAGTACACCTCGCTTTGCCACACCGATCTTTATTTCTGGGAGGCTAAGGACCAGACTCCCTTATTTCCACGCATTTTCGGCCATGAAGCTGCAGGGATTGTTGAAAGTGTTGGTGAGGGT
